# Mechanisms of neural infiltration-mediated tumor metabolic reprogramming impacting immunotherapy efficacy in non-small cell lung cancer

**DOI:** 10.1186/s13046-024-03202-9

**Published:** 2024-10-10

**Authors:** Yuanyuan Zheng, Lifeng Li, Zhibo Shen, Longhao Wang, Xiaoyu Niu, Yujie Wei, Shilong Sun, Jie Zhao

**Affiliations:** 1 Internet Medical and System Applications of National Engineering Laboratory, the First Affiliated Hospital of Zhengzhou University, Zhengzhou, 450052 Henan China; 2 Cancer Center, The First Affiliated Hospital of Zhengzhou University, Zhengzhou, 450052 Henan China; 3 Department of Anesthesiology, the Affiliated Cancer Hospital of Zhengzhou University, Henan Cancer Hospital, Zhengzhou, 450052 Henan China; 4 Department of Pharmacy, the First Affiliated Hospital of Zhengzhou University, Zhengzhou, 450052 Henan China

**Keywords:** Non-small cell lung cancer, Neural infiltration, Tumor microenvironment, 5-hydroxytryptamine, Tumor metabolic reprogramming

## Abstract

**Background:**

Current evidence underlines the active role of neural infiltration and axonogenesis within the tumor microenvironment (TME), with implications for tumor progression. Infiltrating nerves stimulate tumor growth and dissemination by secreting neurotransmitters, whereas tumor cells influence nerve growth and differentiation through complex interactions, promoting tumor progression. However, the role of neural infiltration in the progression of non-small cell lung cancer (NSCLC) remains unclear.

**Methods:**

This study employs the techniques of immunohistochemistry, immunofluorescence, RNA sequencing, molecular biology experiments, and a murine orthotopic lung cancer model to deeply analyze the specific mechanisms behind the differential efficacy of NSCLC immunotherapy from the perspectives of neuro-tumor signal transduction, tumor metabolism, and tumor immunity.

**Results:**

This study demonstrates that nerve growth factor (NGF) drives neural infiltration in NSCLC, and 5-hydroxytryptamine (5-HT), which is secreted by nerves, is significantly elevated in tumors with extensive neural infiltration. Transcriptome sequencing revealed that 5-HT enhanced glycolysis in NSCLC cells. Pathway analysis indicated that 5-HT activated the PI3K/Akt/mTOR pathway, promoting tumor metabolic reprogramming. This reprogramming exacerbated immunosuppression in the TME. Neutralizing 5-HT-mediated metabolic reprogramming in tumor immunity enhanced the efficacy of PD-1 monoclonal antibody treatment in mice.

**Conclusions:**

The findings of this study provide a novel perspective on the crosstalk between nerves and lung cancer cells and provide insights into further investigations into the role of nerve infiltration in NSCLC progression.

**Supplementary Information:**

The online version contains supplementary material available at 10.1186/s13046-024-03202-9.

## Background

Lung cancer is the leading cause of cancer-related incidence and mortality worldwide. According to the latest report from the World Health Organization, there were approximately 2.2 million new cases of lung cancer and 1.8 million deaths from lung cancer globally in 2020, accounting for 18.0% of total cancer-related deaths [1]. Lung cancer is a heterogeneous disease with diverse molecular and pathological characteristics [2]. Non-small cell lung cancer (NSCLC) accounts for approximately 80–85% of all lung cancer types [3]. Only a few patients with NSCLC are diagnosed at an early stage (Stage I or II) when surgical resection is feasible. More than 60% of patients with NSCLC are diagnosed at an advanced stage or with metastasis (Stage III or IV), thereby often missing the opportunity for surgical intervention. This observation indicates that enhancing long-term survival will necessitate the adoption of more effective systemic therapeutic approaches [4]. Immune checkpoint inhibitors (ICIs), particularly programmed cell death protein 1 (PD-1)/programmed death-ligand 1 (PD-L1) inhibitors, have significantly improved treatment outcomes for advanced NSCLC, transforming the therapeutic landscape of this disease over the past decade [5]. However, clinical trials involving nivolumab have shown that PD-L1 expression alone is neither a consistent predictor of therapeutic efficacy nor patient benefit; patients with low PD-L1 expression can still derive substantial benefit from ICI therapy [6, 7]. This highlights the challenge of accurately identifying patients who are most likely to benefit from ICI therapy, leading to potential undertreatment or overtreatment of specific individuals. Therefore, identifying factors that impede the immune response and developing more reliable predictive markers for immunotherapy response are crucial for broadening the applicability of ICIs in NSCLC and maximizing their therapeutic potential.

With the in-depth analysis of the complexity and diversity of the tumor microenvironment (TME), it has been confirmed that multiple biomarkers are highly effective in identifying the responsiveness to ICIs, playing a critical role in predicting and guiding responses to immunotherapy [8]. Interactions between cancer cells and stromal cells within the TME, including vascular endothelial cells, fibroblasts, immune cells, and the extracellular matrix, have been well-characterized, leading to the development of various innovative targeted anti-tumor strategies [9]. Despite its potential significance, the contribution of the nervous system to cancer etiology, particularly the role of nerve fibers within the TME, continues to be a markedly underexplored area in cancer biology research. It is widely recognized that tumors can grow around existing nerves and eventually invade them, a process known as perineural invasion (PNI) [10]. This phenomenon is often linked to poor tumor prognosis, with underlying mechanisms potentially including the provision of survival signals by nerves, reduction in tumor cell apoptosis, and facilitation of tumor metastasis and dissemination [11, 12]. However, this description solely outlines the process of tumor invasion into nerves, implying that nerves are passive participants in tumor progression and that it is insufficient as a perfect prognostic marker. It is only recently, with the advancement of research into neural functions, that numerous studies have revealed parallel mechanisms by which nerves regulate the functions of both normal tissues and tumors. The crosstalk between nerves and cancer cells involves the release of neurotransmitters and neurotrophic factors that stimulate cancer cell growth and dissemination. Until recently, neurogenesis and axon formation in the TME were not considered active contributors to tumor progression [13]. In gastric cancer, cholinergic nerves stimulate the expression of nerve growth factors (NGFs) mediated by the gastric epithelium, promoting tumor cell proliferation and tumorigenesis [14]. In prostate cancer, sympathetic and parasympathetic nerves are involved in tumor growth and metastasis, with proNGF released by prostate cancer cells driving neuronal growth [15, 16]. Moreover, evidence suggests that pancreatic ductal adenocarcinoma (PDAC) cells secrete NGF to promote tumor innervation. Peripheral axons facilitate the growth of PDAC cells by releasing serine [17]. In estrogen receptor-positive breast cancer, NGF inhibits the C2-mediated apoptosis of breast cancer cells by activating the nuclear factor NF-κB and regulates the primary tumor response to tamoxifen [18]. These pioneering studies have advanced the understanding of the underlying cellular and molecular mechanisms, providing insights into neurogenic cancer therapies.

Therefore, the development of various therapeutic approaches in cancer neuroscience warrants further preclinical and clinical exploration. However, there is a lack of research on the degree of nerve infiltration within the TME and its impact on the occurrence and progression of NSCLC. Currently, there is no research that has thoroughly investigated the mechanisms by which nerve infiltration within the TME affects NSCLC progression and the effectiveness of immunotherapy. This study aims to elucidate the molecular mechanisms by which nerve infiltration within the TME regulates tumorigenesis and development, exploring the neuro-tumor-immune regulatory network’s influence on NSCLC treatment and prognosis. This research will provide new theoretical foundations for the diagnosis and treatment of NSCLC and examine the potential for targeting nerve infiltration as a therapeutic strategy for NSCLC.

## Methods

### Cell line culture

Human NSCLC (A549 and H460), normal lung epithelial (BEAS-2B), and mouse Lewis lung cancer (LLC-luciferase) cell lines were obtained from the Shanghai Cell Resource Center Academy of Sciences (Shanghai, China). All cell lines were authenticated using short tandem repeat profiling (QuiCell Biological, Shanghai, China) to confirm the absence of cross-contamination. The cells were cultured in a complete medium comprising Dulbecco’s Modified Eagle Medium (DMEM, No.SH30022.01; HyClone, Logan, UT, USA) supplemented with 1% penicillin-streptomycin (No. C0224; Beyotime, Jiangsu, China) and 10% fetal bovine serum (No. FSP500; Suzhou ExCell Bio, Jiangsu, China). Rat pheochromocytoma cells (PC12) were purchased from Pricella Life Technology Co., Ltd (Hubei, China). The cells were maintained in RPMI-1640 (No.SH30809.01; HyClone) supplemented with 1% penicillin-streptomycin, 15% horse serum (No.S9050; Solarbio, Beijing, China), and 5% fetal bovine serum. PC12 cells gradually formed clusters and maintained a round, undifferentiated phenotype. All cell lines were grown under the same conditions: 37 °C in a 5% CO_2_ incubator. Cells were passaged every 2–3 days, and those in the logarithmic growth phase were analyzed.

### Co-culture and neural axonal growth assays

A 0.1 mg/mL poly-L-lysine solution (No.PB180523; Pricella) was added to a 24-well plate and incubated for 2 h. Subsequently, the poly-L-lysine solution was removed, and the plates were washed thrice with phosphate-buffered saline (PBS) and air-dried in a sterile environment. PC12 cells (5 × 10^^3^/mL) were seeded in 24-well plates and starved for 24 h in DMEM supplemented with 1% horse serum. Tumor cells (A549 and H460) and normal lung epithelial cells (BEAS-2B) were seeded in the upper chamber of a Transwell (12 mm diameter, 0.4 μm pore size) plate at 5 × 10^^3^ cells /well. After 3–5 days of co-culturing, neurite outgrowth of PC12 cells was observed and recorded using a microscope. PC12 cells with neurites at least twice the size of the cell body were considered differentiated.

### Cell proliferation and cell colony-forming unit assay

Cells (A549, H460, and BEAS-2B) treated with 5-hydroxytryptamine (5-HT) for 24 h or co-cultured with PC12 cells for 48 h were seeded in 96-well plates. Upon adherence, 10 µL of cell counting kit 8 (CCK-8) reagent was added to each well and incubated for 1 h at 37 °C. Cell viability was quantified by measuring absorbance at 450 nm using a microplate reader. Cells treated with 0.1% dimethyl sulfoxide (DMSO) served as 5-HT treatment controls. For colony-forming unit analysis, cells were seeded in 6-well plates at 500–1000 cells/well. After adherence, the cells were treated with 5-HT/DMSO or co-cultured with PC12 cells for 7–10 days. After washing with PBS, the cells were fixed with 4% paraformaldehyde (No. P0099; Beyotime) for 15 min, stained with crystal violet solution (No. C0121; Beyotime) for 15 min, and counted after washing.

### Cell invasion and migration assays

Frozen-thawed Matrigel (No. 356230; BD Biosciences, San Jose, CA, USA; 50 µL/cm^2^) was applied to the Transwell chamber (8.0 μm pore size, 24-well insert) and incubated in a cell incubator for 30 min. After 24 h of serum starvation, NSCLC cells were harvested, digested, and resuspended in a serum-free medium before being seeded into the Transwell chambers. The lower section of a 24-well plate was filled with 600 µL of serum-containing medium and reincubated for 24 h. Non-migrated cells in the upper chamber were removed, and the remaining cells were fixed with 4% paraformaldehyde for 15 min and stained with crystal violet for an additional 15 min. Observations and photographic documentation were conducted using an inverted microscope, with randomly selected fields of view captured for statistical analysis. For the migration assay, NSCLC cells in the logarithmic growth phase were retrieved from 6-well plates (1 × 10^^6^ cells/well). After 24 h of serum starvation, cells were mechanically scratched with a 10-µL pipette tip along a straight line, followed by washing with PBS to remove floating cells. Images were captured using an inverted microscope at specific time points for subsequent statistical analysis.

### Cellular energy metabolism assay

The oxygen consumption rate (OCR; 103015-100; Agilent, Santa Clara, CA, USA) and extracellular acidification rate (ECAR; 103020-100; Agilent) were measured using a Seahorse XFe96 extracellular flux analyzer (Seahorse Bioscience, North Billerica, MA, USA). Cells were seeded at 1 × 10^^5^ cells/well in an XFe96 cell culture microplate (Seahorse Bioscience) and cultured overnight until they adhered to the bottom of the plate. Subsequently, the cells were transferred to an assay medium supplemented with 1 mM pyruvate, 2 mM glutamine, and 10 mM glucose for 1 h before the assay and incubated at 37 °C. Following baseline measurements, the OCR was assessed by adding carbonylcyanide-p-trifluoromethoxyphenylhydrazone (FCCP) and rotenone/antimycin A to each well. The ECAR was determined by adding oligomycin and 2-deoxy-D-glucose (2-DG).

### Construction of plasmids

In this study, HTR1D was knocked out using the CRISPR/Cas9 gene editing system. The following primers were synthesized by Eurofins Genomics: HTR1D_sg1-forward (5′-CACCGTAGTTGGA GCTGATGGCGT-3′), HTR1D_sg1-reverse (5′-AAACACGCCATCAGCTCCAATAC-3′), HTR1D_sg2-forward (5′-CACCGCTTCTGGTAGTTGGAGCTGA-3′), and HTR1D_sg2-reverse (5′-AAACTCAGCTCCAACTACCACAAGC-3′). Cloning was performed using lentiCRISPR v2 (plasmid #52961; Addgene, Watertown, MA, USA). All the constructs were confirmed through DNA sequencing.

### Generation of knockdown cell lines

To generate shHTR1D-GFP-A549/H460 cells, lentiviral particles were produced by transfecting 293T cells with target sequences cloned into pLentiCRISPRv2 (52961; Addgene). The medium was changed the following day, and the viral-containing supernatant was collected within 48 h post-transfection, filtered through a 0.45-µm filter (SLHV033RB; MilliporeSigma, Burlington, MA, USA), and subsequently used to infect the target cells with polybrene (8 µg/mL; TR-1003- G; Sigma-Aldrich, Burlington, MA, USA). A549 and H460 cells were infected via incubation with lentivirus-containing supernatant for 48 h. The transduced cells were selected using puromycin (A1113803; Gibco, Grand Island, NY, USA). The efficiency of HTR1D knockdown was analyzed through western blotting.

### Enzyme-linked immunosorbent assay (ELISA)

NGF (No. SEKH0268; Solarbio) in the cell supernatant was quantified using ELISA. The standard, blank, and sample wells were prepared using a microplate reader. Diluted samples were added to designated wells, followed by the addition of 100 µL of the enzyme-labeled reagent to each well. The plates were sealed with membranes and incubated at 37 °C for 60 min. After incubation, the liquid was aspirated, and the wells were washed with washing solution, followed by a brief drying period. Subsequently, 50 µL chromogens A and B were each added to initiate the colorimetric reaction and incubated at 37 °C in the dark for 15 min. The reaction was stopped by adding 50 µL of termination solution to each well, resulting in a color change from blue to yellow. Absorbance at 450 nm was measured using a microplate within 15 min of adding the termination solution.

### RNA isolation and real-time quantitative PCR (RT‒qPCR) analysis

Total RNA was extracted from cells using TRIzol reagent (No. 10296010; Thermo Fisher Scientific, Waltham, MA, USA) following the manufacturer’s protocol. The Revert Aid First Strand cDNA Synthesis Kit (No. AG11706; Accurate Biology, Hunan, China) was used for cDNA synthesis, and the primer sequences are listed in Table 1. RT‒qPCR was conducted using SYBR Green PCR master mix (No. AG11718; Accurate Biology) on a Thermo Fisher 7500 Real-Time PCR system. Amplification conditions included initial denaturation at 95 °C for 5 min, followed by 40 cycles of 95 °C for 10 s and 60 °C for 30 s. Relative gene expression was determined by normalizing target gene expression to that of β-actin. Each experiment was independently replicated at least thrice, and the results were derived from the CT values obtained during RT‒qPCR analysis. Data were analyzed using the 2-ΔΔCt method.

**Table 1:** Primer sequences for validation of gene targets

Gene	Forward primer	Reverse primer
β-actin	CCTTCCTGGGCATGGAGTC	TGATCTTCATTGTGCTGGGTG
NGF	CGCTCATCCATCCCATCCCATTC	CTTGACAAGGTGTGAGTCGTGGT
BDNF	TGGCTGACACTTTCGAACAC	CCTCATGGACATGTTTGCAG
GDNF	GGCAGTGCTTCCTAGAAGAGA	AAGACACAACCCCGGTTTTTG
NT-3	CCGTGGCATCCAAGGTAACAA	GCAGTTCGGTGTCCATTGC
NT-4	CTGTGTGCGATGCAGTCAGT	TGCAGCGGGTTTCAAAGAAGT
TrkA	AACCTCACCATCGTGAAGAGT	TGAAGGAGAGATTCAGGCGAC
TrkB	TCGTGGCATTTCCGAGATTGG	TCGTCAGTTTGTTTCGGGTAAA
TrkC	CTTTGCCCAGCCAAGTGTAGT	CGTGATGTTGATACTGGCGTT
p75^NTR^	ACGGCTACTACCAGGATGAG	TGGCCTCGTCGGAATACGTG
ALDOA	CAGGGACAAATGGCGAGACTA	GGGGTGTGTTCCCCAATCTT
ENO1	GCCGTGAACGAGAAGTCCTG	ACGCCTGAAGAGACTCGGT
HK2	TTGACCAGGAGATTGACATGGG	CAACCGCATCAGGACCTCA
LDHA	TTGACCTACGTGGCTTGGAAG	GGTAACGGAATCGGGCTGAAT
PDK1	CTGTGATACGGATCAGAAACCG	TCCACCAAACAATAAAGAGTGCT
PDK4	GGAAGCATTGATCCTAACTGTGA	GGTGAGAAGGAACATACACGATG
PFKL	GCTGGGCGGCACTATCATT	TCAGGTGCGAGTAGGTCCG
PGAP1	CTGGCAAAACGCTATCCCG	GGAATACCCGTCAAAGGGAGAAT
PGK1	GACCTAATGTCCAAAGCTGAGAA	CAGCAGGTATGCCAGAAGCC
PGK2	CACACCGCGCTCATAGTTC	CTCCACCAAGTATAGCCAGAAAG
PKM	ATAACGCCTACATGGAAAAGTGT	TAAGCCCATCATCCACGTAGA
POGLUT1	GAGGTAGTCAGACGGAAGCTA	GAGGGGAACATGCAGTCATTT

### Transcriptomic analysis

Total RNA was extracted from NSCLC cells treated with 5-HT for 24 h for RNA-sequencing. Three replicates per group were sequenced at BGI (Shenzhen, Guangdong, China). The adopted transcriptomics analysis methods have been described in our previous study [19].

### Protein isolation and immunoblotting

The cells were harvested and lysed in NP40 lysis buffer containing 50 mM Tris-HCl (pH 7.4), 150 mM sodium chloride, 2 mM magnesium chloride, and 0.5% NP40 on ice for approximately 30 min. The cell extracts were centrifuged to remove the insoluble materials. Immunoblotting was performed according to standard protocols. Samples were resolved by 10% or 12% SDS‒PAGE and transferred to PVDF membranes (IPVH00010; Immobilon membranes; MilliporeSigma). Fluorescence-labeled secondary antibodies were used for detection. Proteins were visualized using an enhanced chemiluminescence detection kit (P0018AFT, P0018FS; Beyotime) and an automatic chemiluminescence image analyzer (Tanon5200, Tanon Science & Technology, Shanghai, China).

### Immunofluorescence

Cells in the logarithmic growth phase were seeded into 96-well plates and treated with 5-HT for 24 h. The cells were subsequently washed with PBS and fixed with 4% paraformaldehyde for 20 min at -20 °C. After three washes with PBS, a blocking solution containing bovine serum albumin was added, and the plates were blocked at room temperature for 2–3 h. Primary antibodies (1:200) were applied and incubated overnight at 4 °C. After washing with PBS, an Alexa Fluor 488 conjugated goat Anti-Rabbit IgG (H + L) secondary antibody (green; dilution 1:500) was added and incubated for 2–3 h at room temperature. DAPI (5 µg/mL) was added for nuclear staining and incubated at room temperature in the dark for 20 min. Images were captured using an inverted fluorescence microscope at 200x magnification (Olympus CKX53; Olympus Corporation, Center Valley, PA, USA).

### Orthotopic xenografts

We obtained 6-week-old male C57BL/6 mice weighing 18 ± 2 g from Beijing Vital River Laboratory Animal Technology Co., Ltd., China. Mice were anesthetized using sodium pentobarbital (40 mg/kg). An incision of approximately 5 mm was made 1 cm below the left anterior axillary line, and the skin and subcutaneous tissue were separated to expose the chest wall. LLC-luciferase cells in the logarithmic growth phase were mixed with the matrix gel at a 1:1 ratio, and the cell concentration was adjusted to 1 × 10^^6^/mL. Using an insulin syringe, the cell suspension was inserted vertically into the left lung at a depth of approximately 3 mm. After completing the injection, the incision was sutured, and erythromycin was applied to the suture. In vivo imaging was performed after one week. The mice were intraperitoneally injected with D-luciferin potassium salt at a dosage of 10 µL/g body weight. Subsequently, 10 min post-injection, the anesthetized mice were imaged using an in vivo imaging system. Animals were anesthetized before euthanasia was applied via cervical dislocation, after each experiment or upon reaching a predetermined endpoint (i.e., loss of ≥ 20% body weight or notable deterioration of body condition).

### Mouse model of nerve-infiltrating lung cancer *in situ*

We developed an in situ mouse model of lung cancer. One week after its construction, the mice were injected intraperitoneally with D-fluorescein potassium salt at 10 µL/g body weight, followed by in vivo imaging to confirm the presence of tumor signals in the lungs. Mice were administered NGF (50 ng/mL) via intraperitoneal injection every two days, whereas control mice received an equivalent volume of saline. The tumor size was monitored using in vivo imaging. After 15 days, the mice were euthanized via the spinal cord dislocation method in accordance with the guidelines.

### Functional assay of infiltrating CD8+ T cells in mouse tumors

Immediately after excising mouse tumor tissues, they were placed on ice and rinsed with pre-cooled PBS (pH 7.4) at 4 °C. Tumor tissues were minced, and 2 mL of collagenase IV (1 UI/mL) was added and fully digested for 2 h at 37 °C. The resulting tissue suspension was sequentially filtered through a 200-mesh sieve to remove undigested tissue and debris and centrifuged at 300 × *g* for 5 min; this process was repeated thrice. Subsequently, resuspend the cells in 5 mL of RPMI-1640 medium and carefully layered the cell suspension over 5 mL of Ficoll-Paque PLUS in a 15 mL conical tube. Centrifuged at 400 × *g* for 30 min. After centrifugation, carefully collect the lymphocyte-enriched interface (the white, cloudy layer) using a sterile pipette and transfer it to a new 15 mL conical tube. Wash the collected cells by adding 5 mL of PBS, followed by centrifugation at 300 x g for 10 min. Aspirate the supernatant carefully, ensuring the cells remain undisturbed. Finally, the cell is resuspended for subsequent analysis or culture.

Surface antibodies were added to the resuspended cells, which were incubated for 15 min at 4 °C and protected from light. After fixation, the cells were centrifuged, the supernatant was discarded, and the cells were permeabilized with a 100× membrane permeabilization agent. Following another 30 min centrifugation, the supernatant was discarded, internal factor antibodies were added, and the cells were incubated at 4 °C and protected from light for 15 min. Subsequently, the cells were analyzed through flow cytometry.

### Immunohistochemistry (IHC)

The expression of the pan-neuronal marker PGP9.5 (protein gene product 9.5/UCH-L1/ PARK5) was assessed in neoplastic cells and peritumoral tissue. The 85 pairs of human NSCLC tissue samples were obtained from the First Affiliated Hospital of Zhengzhou University. Tumor tissues were dehydrated and treated with peroxidase. Antigen retrieval was performed in a pressure cooker using 0.01 mol/L citrate buffer (pH 6.0). Following the manufacturer’s instructions, the samples were incubated with the PGP9.5 antibody (dilution: 1:1000) overnight at 4 °C. Subsequently, the slides were stained using the Histostain-Plus kit (SP-9000) and 3.3-diaminobenzidine tetrahydrochloride (DAB; ZLI- 9032; ZSGB-BIO, Beijing, China). Finally, the nuclei were stained with hematoxylin. Two senior pathologists independently scored the IHC results in a double-blind manner. Proportion scores were assigned as follows: 0 for 0‒5% of tumor cells exhibiting positive staining, 1 for 6‒25%, 2 for 26–50%, 3 for 51–75%, and 4 for 76–100%. The staining intensity was scored on a scale of 0–3 as follows: 0, negative; 1, weak; 2, moderate; and 3, strong. The overall positive grade of IHC was determined by combining the proportion of positive tumor cells and staining intensity: 0 was classified as negative (-), 1–3 as weakly positive (+), 4–5 as positive (++), and 6–7 as strongly positive (+++). Survival analysis was conducted based on PGP9.5 expression, with low expression defined as (-) and (+) and high expression defined as (++) and (+++).

### Antibodies and reagents

The primary antibodies used in this study were as follows: anti-p-phosphoinositide-3-kinase (PI3K), anti-c-Myc, anti-PGP9.5 (ab52918; Abcam, Cambridge, UK), anti-5-HT (No. 20080; Immunostar, Hudson, WI, USA), anti-PD-L1 (No. 66248; Proteintech, Wuhan, China), anti-human leukocyte antigen class I ABC (No. 15240; Proteintech), anti-p-AKT (Ser473; No. 66444, Proteintech), anti-AKT (No. 10176; Proteintech), anti-mammalian target of rapamycin (mTOR; No. 66888; Proteintech), anti-p-mTOR (No. 67778; Proteintech), anti-5HT1D (No. DF2706; Affinity Biosciences, London, England), anti-PI3K (No. 133595; Abcam), anti-hexokinase II (No. 209847; Abcam), anti-HIF1α (No. 179483; Abcam), and anti-p-PI3K (p85/p55; No. 4228; CST, Danvers, MA, USA). GAPDH (Hangzhou Goodhere Biotechnology, Hangzhou, China) was used as a loading control. The secondary antibodies, peroxidase-conjugated goat anti-mouse IgG and peroxidase-conjugated goat anti-rabbit IgG, were purchased from ZGSB.Bio, Inc., (Beijing, China). Additional reagents included 2-DG (HY-13966), PKI-402 (HY-10683), rapamycin (HY-0219), nivolumab (HY-P9903), and serotonin (No. HY-B1473A) were purchased from Med Chem Express (MCE; Monmouth Junction. NJ, USA). D-Luciferin (No. ST196) potassium salt was purchased from Beyotime, and a CCK-8 (No. IV08-500) was purchased from Invigentech (Irvine, CA, USA).

### Quantification and statistical analysis

All the data were obtained from at least three independent experiments. Cell colony-forming units were quantified by counting the number of cells in each dish using the ImageJ software. The data were presented as the mean ± standard deviation (SD), with error bars representing SD from three independent experiments. Data visualization and descriptive statistics were performed using GraphPad Prism version 8. The significance of differences between the two groups was assessed using Student’s *t-test*. Survival curves were generated using the Kaplan‒Meier method. Differences among multiple groups were analyzed using one-way ANOVA followed by Tukey’s multiple comparison test. A two-tailed *p-value* < 0.05 was considered to indicate statistical significance in all cases, *p < 0.05, **p < 0.01, and ***p < 0.001. * indicates a statistically significant difference compared with the control group; # indicates a statistically significant difference compared with the 5-HT group.

## Results

### Neural infiltration correlates with prognosis, lymph node metastasis status, pathological grade, and PD-L1 expression in patients with NSCLC

**Fig. 1:**
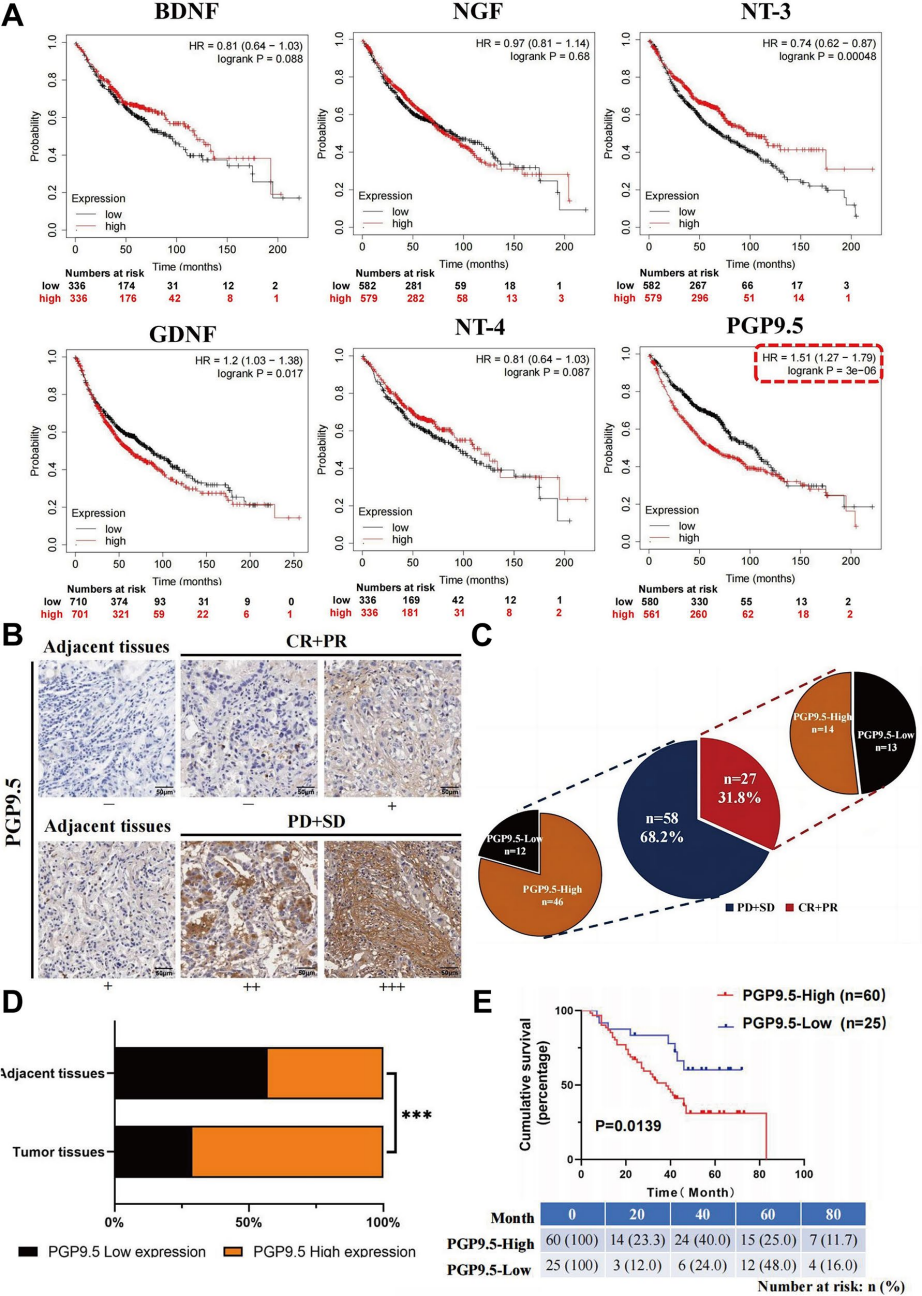
Neural infiltration promotes the development and progression of NSCLC **(A)** The TCGA database was used to screen neuro markers to evaluate the prognosis of non-small cell lung cancer **(**NSCLC). **(B)** Immunohistochemistry (IHC) staining for PGP9.5 was conducted on 85 pairs of NSCLC tissues, and representative images were presented, ranging from the weakest (-, Group 1) to the strongest (+++, Group 4), based on increasing staining intensity. Representative staining results were shown for the effective (CR + PR) and progressive (PD + SD) groups. **(C)** The degree of tumor tissue and paraneoplastic nerve infiltration in patients with NSCLC was statistically analyzed. **(D)** Statistical analysis of the degree of tumor tissue and paraneoplastic nerve infiltration in patients with NSCLC. **(E)** The impact of protein gene product 9.5 (PGP9.5) expression level on survival in patients with NSCLC was assessed, revealing that high PGP9.5 expression was correlated with a worse prognosis compared with low PGP9.5 expression (*p* = 0.028)

Neural markers highly expressed in NSCLC and related to survival were screened using an online database (www.kmplot.com) [20]. The results indicated that the pan-neuronal marker PGP9.5 (UCH-L1/ PARK5) was significantly correlated with the prognosis of patients with NSCLC (*P* < 0.05; Fig. 1A). The PGP9.5 is a developmentally regulated neuron- and neuroendocrine cell-specific ubiquitin carboxy-terminal hydrolase 1 expressed in the mammalian central and peripheral nervous systems [20, 21]. This study comprised 85 patients with NSCLC and complete clinical data. The patients were categorized as responders or non-responders based on their treatment response. Tissue samples were stained with PGP9.5 to evaluate the degree of nerve invasion. Based on the IHC scores, the patients were divided into PGP9.5 high-expression (IHC score of 4–7) and low-expression (IHC score of 0–3) groups (Fig. 1B). The expression levels of PGP9.5 were significantly higher in the non-responder group than in the responder group (46 vs. 14, *p* = 0.0199; Fig. 1C). Nerve invasion was more pronounced in the tumor tissues than in the adjacent tissues (Fig. 1D). Patients with NSCLC were followed-up for a median of 42 months, during which 55.3% (47 patients) died, and 44.7% (38 patients) survived. An analysis of the relationship between PGP9.5 expression and patient survival suggested that neural infiltration is related to overall survival (OS; *p* = 0.0139; Fig. 1E). Furthermore, the chi-square test of clinical parameters revealed that PGP9.5 expression was correlated with lymph node metastasis (*p* = 0.0017), pathological grade (*p* = 0.0262), and PD-L1 expression (*p* = 0.0201; Table 2). The result demonstrated that an elevated expression of the neural marker PGP9.5 in NSCLC is significantly associated with poor prognosis and heightened nerve invasion, and this shows a strong correlation with lymph node metastasis, pathological grade, and PD-L1 expression.

**Table 2:** Association between the expression of PGP9.5 and clinicopathological parameters in NSCLC

Parameter	PGP9.5 Expression		*P*-value
	Low (0–1)	High (2–3)	
All case Efficacy Effective (CR + PR) Progressive (PD + SD) Gender	25 (29.4%) 13 (48.1%) 12 (20.7%)	60 (70.7%) 14 (51.9%) 46 (79.3%)	0.0199^a^ 0.9999
Male	14 (29.8%)	33 (70.2%)	
Female	11 (28.9%)	27 (71.1%)	
Age			0.9999
<63	13 (29.5%)	31 (70.5%)	
≥ 63 Lymphatic node transfer No Yes	12 (29.3%) 20 (44.4%) 5 (12.5%)	29 (70.7%) 25 (55.6%) 35 (87.5)	0.0017^a^
Pathological grading			0.0262^a^
I	2 (66.7%)	1 (33.3%)	
II	21 (35.0%)	39 (65.0%)	
III Tumor size (T) 1 2 3 4	2 (9.1%) 9 (40.9%) 14 (32.6%) 0 (0%) 2 (25.0%)	20 (90.9%) 13 (59.1%) 29 (67.4%) 12 (100%) 6 (75.0%)	0.0828
EGFR			0.6494
Positive	18 (28.1%)	46 (71.9%)	
Negative PD-L1 Positive Negative	7 (33.3%) 13 (21.7%) 12 (48.0%)	14 (66.7%) 47 (78.3%) 13 (52.0%)	0.0201^a^

EGFR: Epidermal growth factor receptor. PD-L1: Programmed cell death ligand 1

a: Statistically significant p-values ( *p* < 0.05 using the chi-square test or Fisher exact tests, if appropriate)

### Crosstalk between NSCLC cells and neuron-like cells

**Fig. 2:**
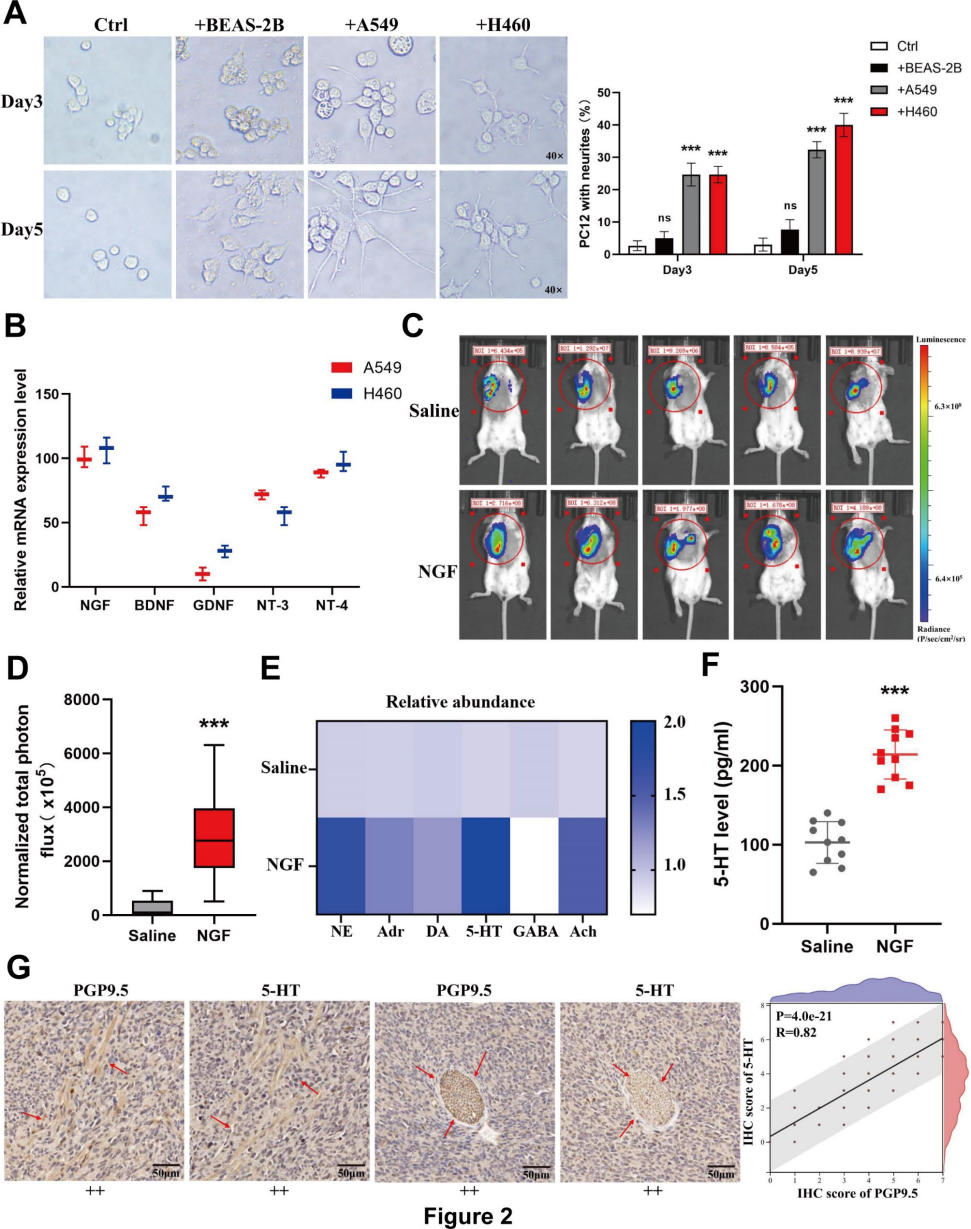
NSCLC secretes NGF to promote axon formation, with nerve invasion degree positively correlated with 5-HT secretion. **(A)** Co-culture experiments were performed over 3–5 days in Transwell Boyden chambers, with NSCLC cells in the upper membrane and the neuronal PC12 cells in the lower membrane. Quantitative statistics of axonal growth in nerve cells showed that PC12 had neurites at least twice the size of the cell body that were considered differentiated. **(B)** Expression of nerve growth factor (NGF), brain-derived neurotrophic factor (BDNF), glial cell line-derived neurotrophic factor (GDNF), and neurotrophin-3/4 (NT-3/4) at the mRNA level in NSCLC cell lines. **(C)** Representative in vivo imaging results of tumor formation in the two mouse groups. **(D)** Statistical graph of tumor fluorescence intensity in two mouse groups. **(E)** Detection of neurotransmitter levels in mouse tumors using ELISA. **(F)** Detection of 5-hydroxytryptamine (5-HT) levels in tumor tissues of two mouse groups using ELISA. **(G)** Tumor tissues from mice were i-serially sectioned and examined for PGP9.5 and 5-HT expression through IHC

To verify the neurotrophic activity of NSCLC cells, we co-cultured NSCLC (A549 and H460) and normal lung epithelial (BEAS-2B) cells with neuron-like cells (PC12) in Transwell plates. Quantitative comparison of PC12 cells with neurites revealed that A549 and H460 cells markedly induced the neuronal differentiation of PC12 cells compared with that of BEAS-2B cells. However, no significant differentiation or axonal outgrowth was observed when PC12 cells were cultured alone (Fig. 2A). These data suggest that NSCLC cells exhibit a neurotrophic activity that induces neuronal differentiation and axonal outgrowth. Subsequently, we added PC12 cell culture supernatant (filtered with a 0.22-µm filter) to culture dishes containing adherent NSCLC and BEAS-2B cells to observe the tumor-promoting effect of nerve cells on tumor cells and observed that the proliferative ability of tumor cells after 48 h of culturing in the PC12 cell culture supernatant was significantly greater than that in standard medium (see Supplementary Fig. 1A, Additional file 1). The colony formation assay indicated that nerve cells improved the survival rate of tumor cells (shown in Supplementary Fig. 1B, Additional file 1). Additionally, Transwell and scratch assays demonstrated that neural cells promoted the invasion (see Supplementary Fig. 1C, Additional file 1) and migration (shown in Supplementary Fig. 1D, Additional file 1) abilities of NSCLC cells. Therefore, co-culturing NSCLC cells with PC12 cells induced neuronal differentiation in PC12 cells, and the presence of nerve cells enhanced the proliferation, invasion, and migration of NSCLC cells.

### NGF promotes axon formation in NSCLC cells

We selected a range of neurotrophic factors known to enhance the survival, differentiation, and functional maintenance of neural cells, including NGF, brain-derived neurotrophic factor (BDNF), glial cell line-derived neurotrophic factor (GDNF), neurotrophin-3 (NT-3), and neurotrophin-4 (NT-4) [22], and evaluated their expression levels in NSCLC cells. The results demonstrated a pronounced upregulation of NGF expression (Fig. 2B). ELISA confirmed that NGF levels were significantly elevated in the supernatants of A549 and H460 cells compared with those in BEAS-2B cells (see Supplementary Fig. 1E, Additional file 1). Furthermore, examination of the expression of neural receptors in PC12 cells revealed that the mRNA levels of TrkA, a receptor for NGF, were significantly higher in PC12 cells within the co-culture system than in normal PC12 cells (see Supplementary Fig. 1F, Additional file 1). When an NGF inhibitor (Ro 08-2750) was added to the co-culture system, the axonal growth of PC12 cells was significantly inhibited, and the neurotrophic activity of NSCLC cells was markedly reduced (shown in Supplementary Fig. 1G, Additional file 1). To further investigate this, we created a neuroinvasive in situ lung cancer model by injecting NGF into mice with seeded tumors. Tumor samples collected at the end of the experiment were subjected to IHC, which showed that the tumors in the NGF-treated group were significantly larger than those in the control group (Fig. 2C, D). Additionally, the expression of neuronal markers PGP9.5 was significantly upregulated (see Supplementary Fig. 2A, Additional file 2), as were proliferative indexes Ki-67 (Supplementary Fig. 2B) and Neural cadherin (N-cadherin) (shown in Supplementary Fig. 2C, Additional file 2), compared with those in the control group. Collectively, NGF levels were significantly elevated in NSCLC cells compared to normal lung epithelial cells, and their interaction with TrkA receptors in PC12 cells enhanced axonal growth and differentiation, promoting tumor growth in a murine model and augmenting neurotrophic activity.

### Nerve infiltration is positively correlated with the neurotransmitter 5-HT

The changes in the expression of neurotransmitters [norepinephrine (NE), adrenaline (Adr), dopamine (DA), 5-HT, gamma-aminobutyric acid (GABA), and acetylcholine (Ach)] in the tumors of mice treated with NGF are shown in Fig. 2E. Quantitative assays revealed that 5-HT levels were significantly elevated in the tumors of mice in the NGF group (Fig. 2F). Additionally, IHC staining demonstrated that high 5-HT expression was correlated with high PGP9.5 expression (Fig. 2G). Based on these findings, we hypothesized that neural infiltration in the TME is involved in 5-HT-mediated tumor progression, prompting further investigation into the biological role of 5-HT in NSCLC.

### 5-HT exhibits a protective effect on NSCLC cells through the HTR1D receptor

**Fig. 3:**
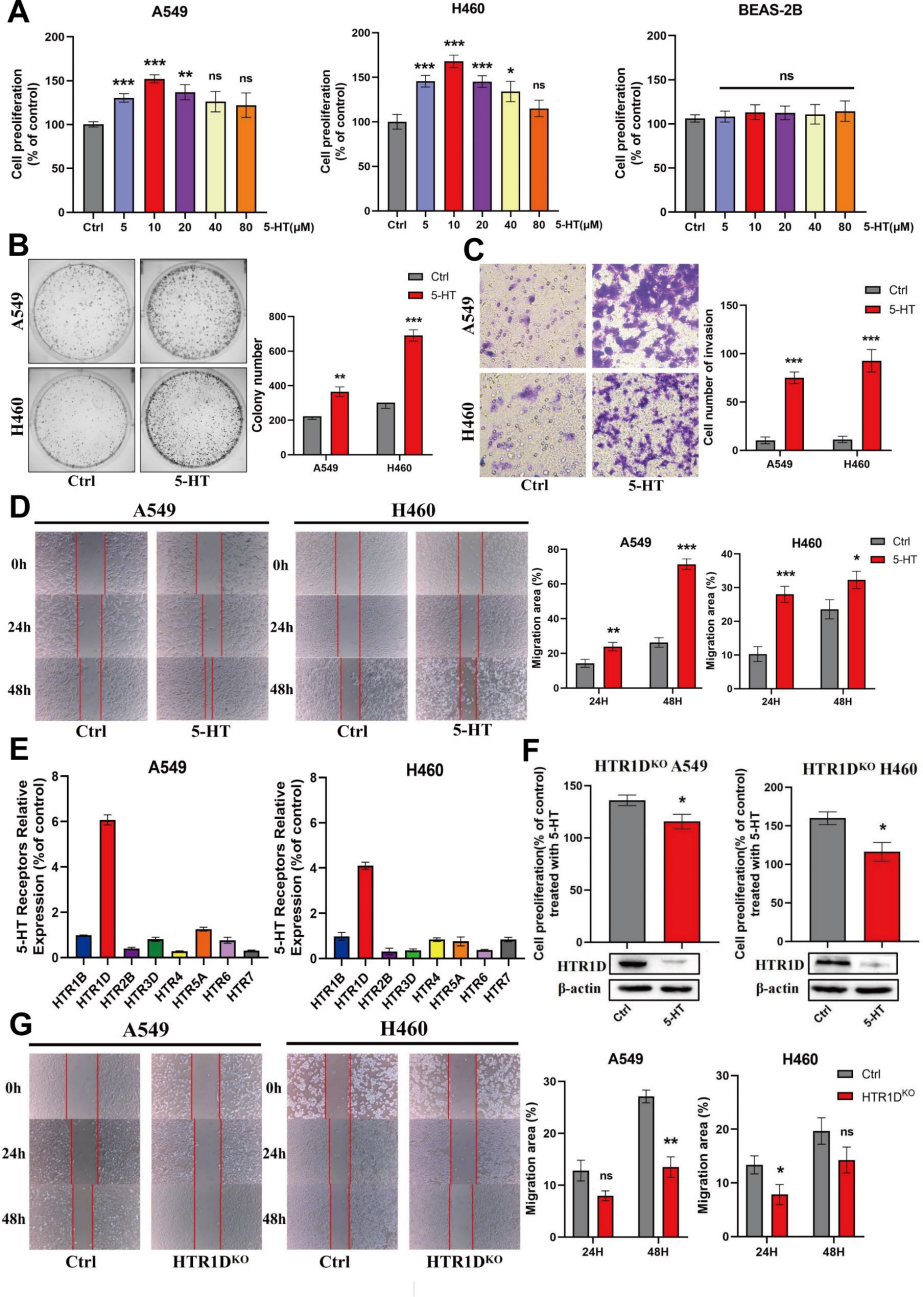
5-HT exhibits a protective effect on NSCLC cells through the HTR1D receptor **(A)** Effect of 5-HT on NSCLC and normal lung epithelial cell viability. Cells were treated with dimethyl sulfoxide (DMSO) or 5-HT for 24 h, and cell viability was detected using the cell counting kit-8 kit. **(B)** Effect of 5-HT on colony formation in NSCLC cells. **(C)** The effect of 5-HT on the invasion of NSCLC cell lines. **(D)** The effect of 5-HT on the migratory ability of NSCLC cell lines. (E) The expression of 5-HTR at the mRNA level in NSCLC cell lines stimulated with 5-HT. **(F)** Effect of 5-HT on the proliferation of HTR1D-knockout cells. **(G)** Effect of 5-HT on the migration of HTR1D^KO^ NSCLC cells. The t-test in GraphPad Prism8 software was used to assess the statistical significance of differences between groups in panels. * The difference between the two groups was significant (*p* < 0.05). Data represent at least three independent experiments (*n* = 3; error bars, SD)

To substantiate the specific impact of 5-HT on tumor progression, we conducted a proliferation assay that demonstrated enhanced proliferation of NSCLC cells following treatment with 10 µM 5-HT for 24 h. In contrast, no significant changes in BEAS-2B cell proliferation were observed under the same conditions (Fig. 3A). In addition, 5-HT promoted colony formation (Fig. 3B), invasion (Fig. 3C), and migration (Fig. 3D) of NSCLC cells.

To identify the receptor subtypes responsible for the tumor-promoting effects of 5-HT, we assessed the mRNA expression of 5-HT receptors in NSCLC cells using RT‒qPCR. The results indicated that HTR1D is a potential target receptor subtype of 5-HT (Fig. 3E). To further validate this hypothesis, we generated A549 (HTR1D^KO^ A549) and H460 (HTR1D^KO^ H460) cell lines with a stable knockout of HTR1D using the CRISPR/Cas9 gene-editing technology. The gene validation results for the HTR1D-knockout cell lines are shown in Supplementary Fig. 2D, Additional file 2. Subsequent experiments revealed that the pro-proliferative (Fig. 3F) and migratory effects (Fig. 3G) of 5-HT were significantly diminished after stimulation with 5-HT for 24 h in the HTR1D-knockout cell lines. These findings imply that HTR1D is the primary receptor subtype through which 5-HT exerts protumorigenic effects. Altogether, 5-HT treatment enhanced NSCLC cell proliferation, colony formation, invasion, and migration primarily through the HTR1D receptor, as evidenced by diminished effects in HTR1D-knockout cell lines.

### Transcriptomics-based analysis of 5-HT regulatory pathways in NSCLC cells

**Fig. 4:**
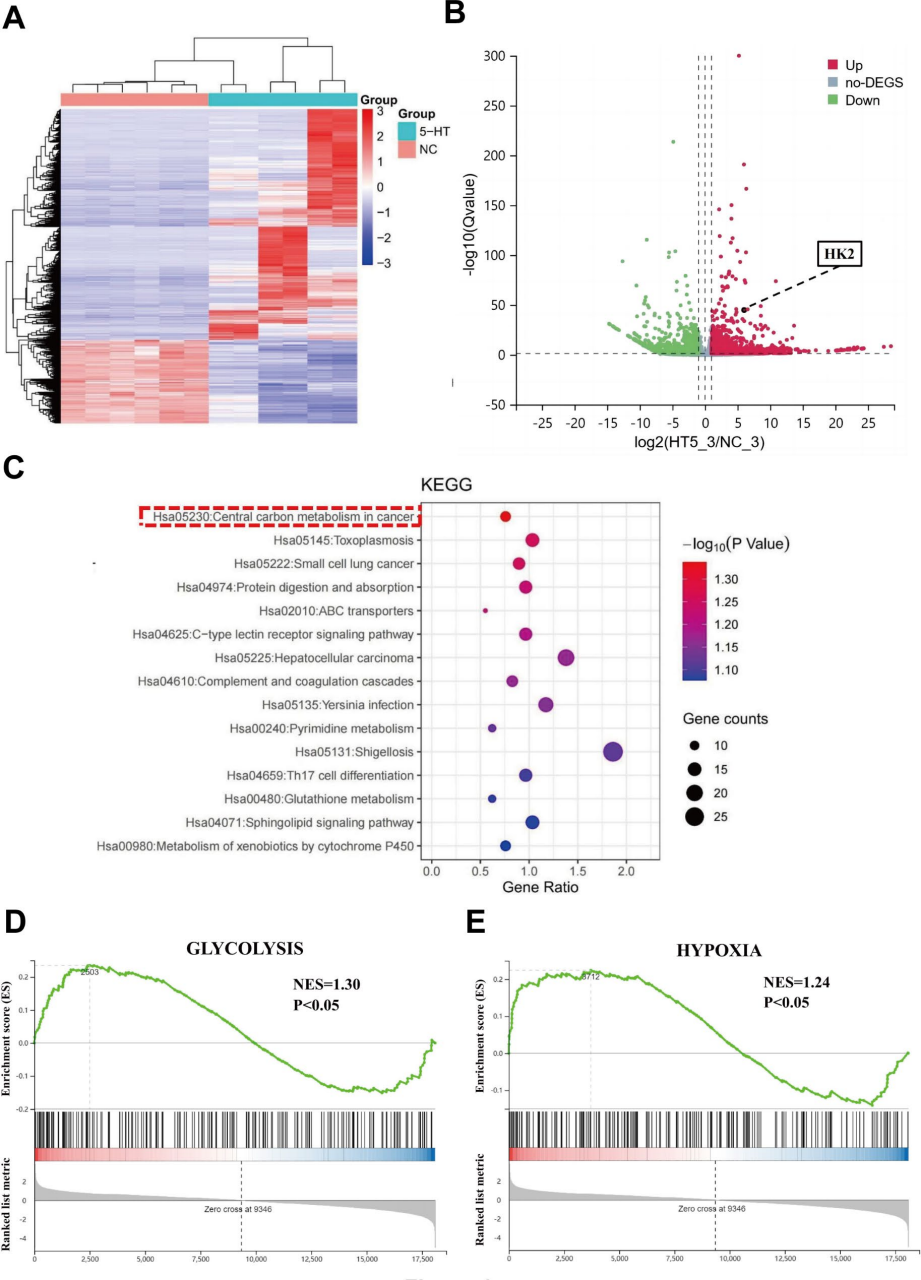
Transcriptome analysis reveals significant enrichment of glycolytic pathways in NSCLC cells after 5-HT action **(A)** Heat map showing the differentially expressed genes (DEGs) modulated by treatment with 5-HT. **(B)** Differential metabolite volcano plots of the control and 5-HT-treated groups. **(C)** Pathway enrichment analysis of DEGs shows a significant enrichment of central carbon metabolism in cancer. **(D)** Gene set enrichment analysis plots based on the gene expression profiles of NSCLC cells. NES represents the normalized enrichment score

To explore the molecular mechanisms underlying the protumorigenic effects of 5-HT, we conducted genome-wide transcriptome sequencing of A549 and H460 cells treated with 5-HT for 24 h. A total of 3,632 differentially expressed genes (DEGs) were identified, comprising 2,671 upregulated and 961 downregulated genes, meeting the criteria of at least a two-fold change and a *p*-value < 0.05 (Fig. 4A). Subsequently, using Q value ≤ 0.05 and |log2(FC) | ≥1 as filtering thresholds, we identified significantly altered metabolic pathways, with particular focus on hexokinase 2 (HK2; Fig. 4B). Functional annotation and pathway analysis of the DEGs were performed using the Kyoto Encyclopedia of Genes and Genome database. The enriched pathways primarily included those related to the central carbon metabolism pathway in cancer (Fig. 4C). Gene set enrichment analysis using hallmark gene sets highlighted substantial alterations in metabolic processes such as glycolysis (*p* < 0.01) and hypoxia pathways (*p* < 0.05; Fig. 4D).

### 5-HT promotes tumor progression by enhancing the Warburg effect in NSCLC cells

**Fig. 5:**
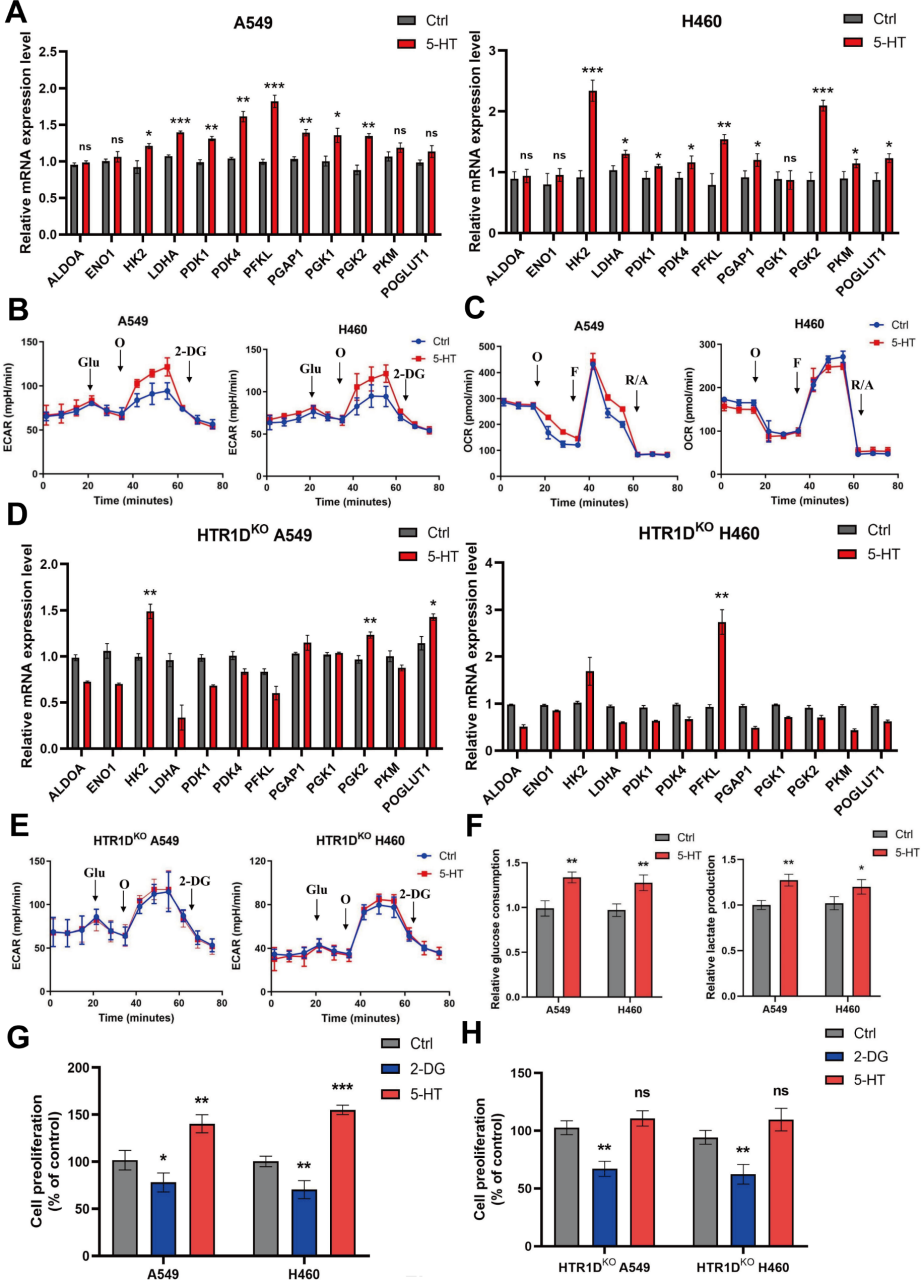
5-HT promotes the survival of NSCLC cells by enhancing the Warburg effect **(A**) The mRNA levels of glycolysis-related enzymes detected in the presence or absence of 5-HT treatment. **(B)** Ratio of extracellular acidification rate (ECAR) in NSCLC cell lines after 5-HT stimulation. Glu, glucose; O, Oligomycin; 2-DG, 2-deoxyglucose. **(C)** The ratio of oxygen consumption rate (OCR) of NSCLC cell lines after 10µM 5-HT stimulation. F, FCCP (carbonyl cyanide-p-trifluoromethoxyphenylhydrazone); R/A, Rotenone/Antimycin A. **(D)** Changes in mRNA levels of glycolysis-related enzymes in HTR1D^KO^ NSCLC cell lines after 10 µM 5-HT stimulation. **(E)** ECAR ratio in HTR1D^KO^ NSCLC cells after 5-HT stimulation. **(F)** Changes in cellular glucose consumption (left panel) and lactate production (right panel) in NSCLC cells upon 10 µM 5-HT stimulation. **(G)** Effect of glycolytic inhibitor (2-DG) on the proliferation of NSCLC cells stimulated by 10 µM 5-HT. **(H)** Effect of 2-DG on the invasiveness of HTR1D^KO^ NSCLC cells stimulated with 10 µM 5-HT

To elucidate the correlation between 5-HT and enhanced glycolytic flux in NSCLC cells, we analyzed the expression of glycolysis-related enzymes in cells treated with 5-HT for 24 h. These enzymes were significantly upregulated at the mRNA level (Fig. 5A). To further investigate the regulatory effects of 5-HT on reprogrammed glycolysis metabolism, ECAR and OCR were also assessed. Our findings demonstrated that 5-HT significantly enhanced the glycolytic capacity of NSCLC cells without notably impacting oxidative phosphorylation (OXPHOS; Fig. 5B, C). This finding suggests that 5-HT predominantly enhances the glycolytic pathway of glucose metabolism in NSCLC cells, which is a characteristic of the “Warburg” effect. Compared to HTR1D non-knockout cells, no significant changes in the mRNA levels of glycolysis-related enzymes were observed in HTR1D-knockout cell lines subjected to 5-HT stimulation (Fig. 5D). Additionally, the cellular energy metabolism assay indicated that cells lacking HTR1D expression showed no significant changes in ECARs after stimulation with 5-HT (Fig. 5E). The assessment of glucose uptake and extracellular lactate levels in 5-HT-treated cells revealed that 5-HT increased glucose consumption and significantly increased lactate production (Fig. 5F). To further confirm that the effect of 5-HT on NSCLC cells is mediated through the modulation of the Warburg effect, we used 2-DG to inhibit glycolysis, compelling the cells to rely on OXPHOS. Inhibition of glycolytic capacity prevents 5-HT from exerting pro-proliferative effects on NSCLC cells, indicating that the protective effect of 5-HT necessitates enhanced aerobic glycolysis (Fig. 5G). Similarly, the absence of the HTR1D receptor attenuated the pro-proliferative effects of 5-HT (Fig. 5H). Our results indicate that the protumorigenic effect of 5-HT is mediated by the enhancement of the Warburg effect in tumor cells.

### 5-HT enhances the Warburg effect by activating the PI3K/Akt/mTOR pathway

**Fig. 6:**
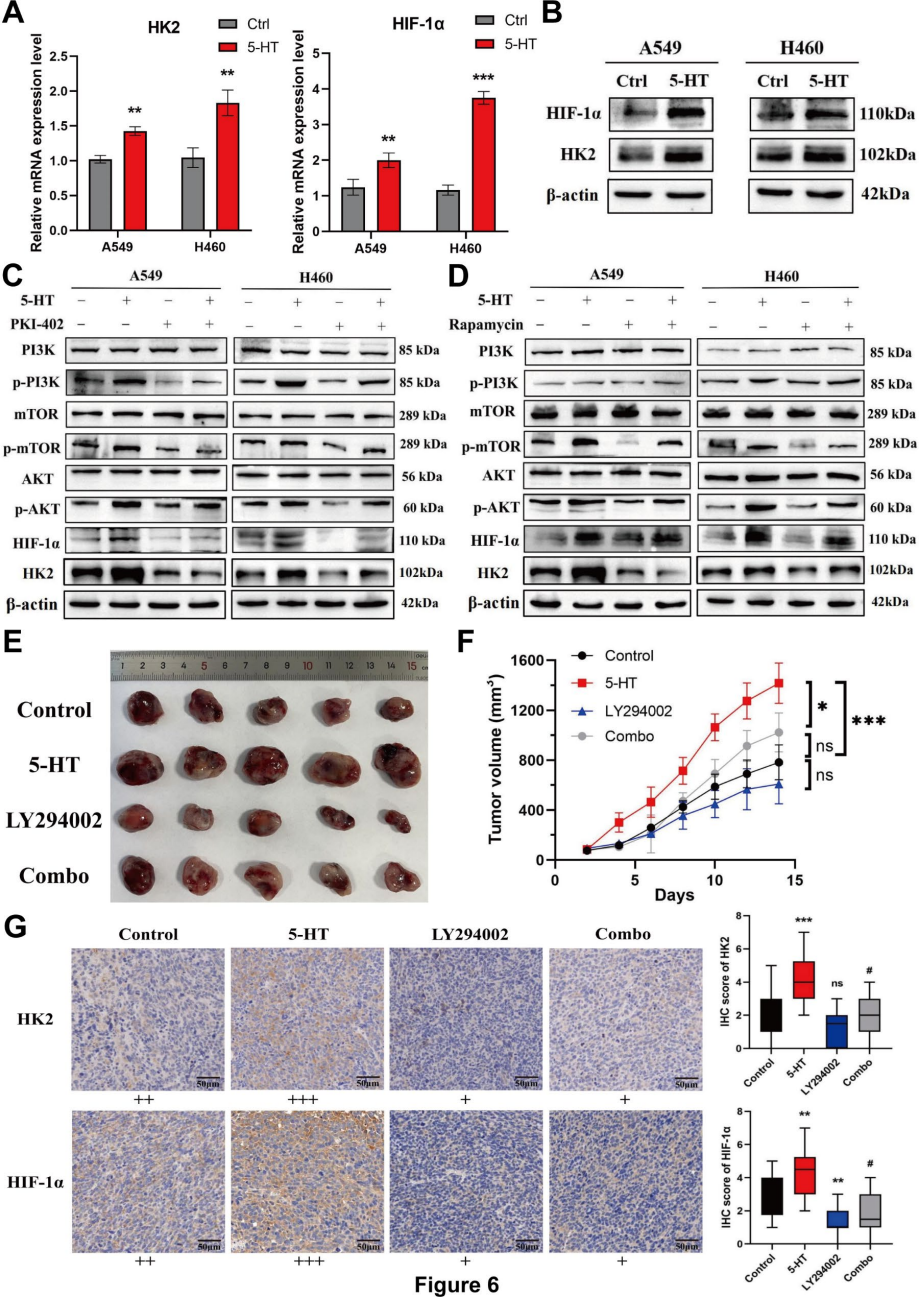
5-HT-mediated metabolic reprogramming is via the activation of the PI3K/Akt/mTOR pathway **(A)** The mRNA expression of hexokinase (HK2) and hypoxia-inducible factor 1-alpha (HIF-1α) was altered under the stimulation of 5-HT. **(B)** Changes in the expression of HK2 and HIF-1α protein under the stimulation of 5-HT. **(C)** Effect of LY294002 (10µM) on 5-HT-mediated PI3K/Akt/mTOR signaling pathway activity. **(D)** Effect of rapamycin (50nM) on 5-HT-mediated PI3K/Akt/mTOR signaling pathway activity. **(E)** Planting of the miceLLC into the dorsal skin of the C57 mice. When the tumor reached 100 mm^3^, 5-HT (45ng/mm^3^, intratumorally, q2d ) and LY294002 (25 mg/kg, ip, q2d) treatments were initiated. The mice were sacrificed on the 21st day of tumor formation, and the tumor was exfoliated and photographed. **(F)** Measure the tumor volume every 3 days. Evaluation of the difference between the size of mice tumors in different therapeutic groups through the t-test with GraphPad Prism8 software. **(G)** IHC of tumor tissue in mice. IHC was performed on tumor tissues of mice and classified according to the degree of staining, and reprehensive pictures were captured. Light yellow indicates weak positivity (+), brownish yellow indicates moderate positivity (++), and brownish-black indicates strong positivity (+++). Statistical plots of HK2 and HIF-1α expression in each group are shown on the right panel. * indicates that compared with the control group, *p* < 0.05, the difference was statistically significant; # indicates that compared with the 5-HT group, *p* < 0.05, the difference is statistically significant)

HK2 is a rate-limiting enzyme in the glycolytic pathway that regulates the rate of glycolysis within cells [23]. In solid tumors, tumor cells frequently experience hypoxia, leading to the lack of oxygen-dependent hydroxylation and proteasomal degradation of hypoxia-inducible factor 1-alpha (HIF-1α), which causes its accumulation. This accumulation initiates the transcription of genes encoding glucose transporters and glycolytic enzymes, thereby elevating glycolytic flux [24]. We found that HK2 and HIF-1α expressions in NSCLC cells were significantly upregulated by 5-HT at the mRNA and protein levels (Fig. 6A, B). Hyperactivation of the PI3K/Akt/mTOR signaling cascade is prevalent in tumors and is critical for cell proliferation, growth, survival, and metabolic reprogramming [25]. Notably, HIF1α is a direct downstream target of mTOR [26, 27]. In NSCLC cells stimulated with 5-HT, we observed a significant upregulation of phosphorylated PI3K (p-PI3K), Akt (p-AKT), and mTOR (p-mTOR; see Supplementary Fig. 2E, Additional file 2). However, in HTR1D-knockdown NSCLC cells, the 5-HT-induced upregulation of p-PI3K, p-AKT, and p-mTOR was not evident (see Supplementary Fig. 2F, Additional file 2). Subsequent rescue experiments using specific PI3K (LY294002, 10 µM) and mTOR (rapamycin, 50 nM) inhibitors demonstrated that the 5-HT-induced phosphorylation of proteins in the PI3K/Akt/mTOR pathway and HIF-1α and HK2 upregulation were effectively inhibited (Fig. 6C, D). This intervention also reversed the 5-HT-induced upregulation of glycolysis-related enzymes (shown in Supplementary Fig. 2G, Additional file 2). In addition, PI3K/ mTOR inhibitors attenuated the 5-HT-induced increase in ECAR (see Supplementary Fig. 3A, Additional file 3), glucose depletion, and lactate production (see Supplementary Fig. 3B, C, Additional file 3). In vivo, the tumor volume in the 5-HT-treated group was significantly larger than that in the control group, whereas treatment with the PI3K inhibitor significantly reduced the tumor burden (Fig. 6E, F). IHC showed that 5-HT treatment upregulated HK2 and HIF-1α expression in tumor tissues, which was reversed by PI3K inhibitor treatment (Fig. 6G). Similarly, treatment with a PI3K inhibitor decreased the phosphorylation of the downstream molecules Akt and mTOR (shown in Supplementary Fig. 3D, Additional file 3). These findings suggest that 5-HT promotes tumor progression by enhancing the Warburg effect via the activation of the PI3K/Akt/mTOR pathway in NSCLC cells.

### Inhibition of metabolic reprogramming synergistically enhances immunotherapy efficacy

**Fig. 7:**
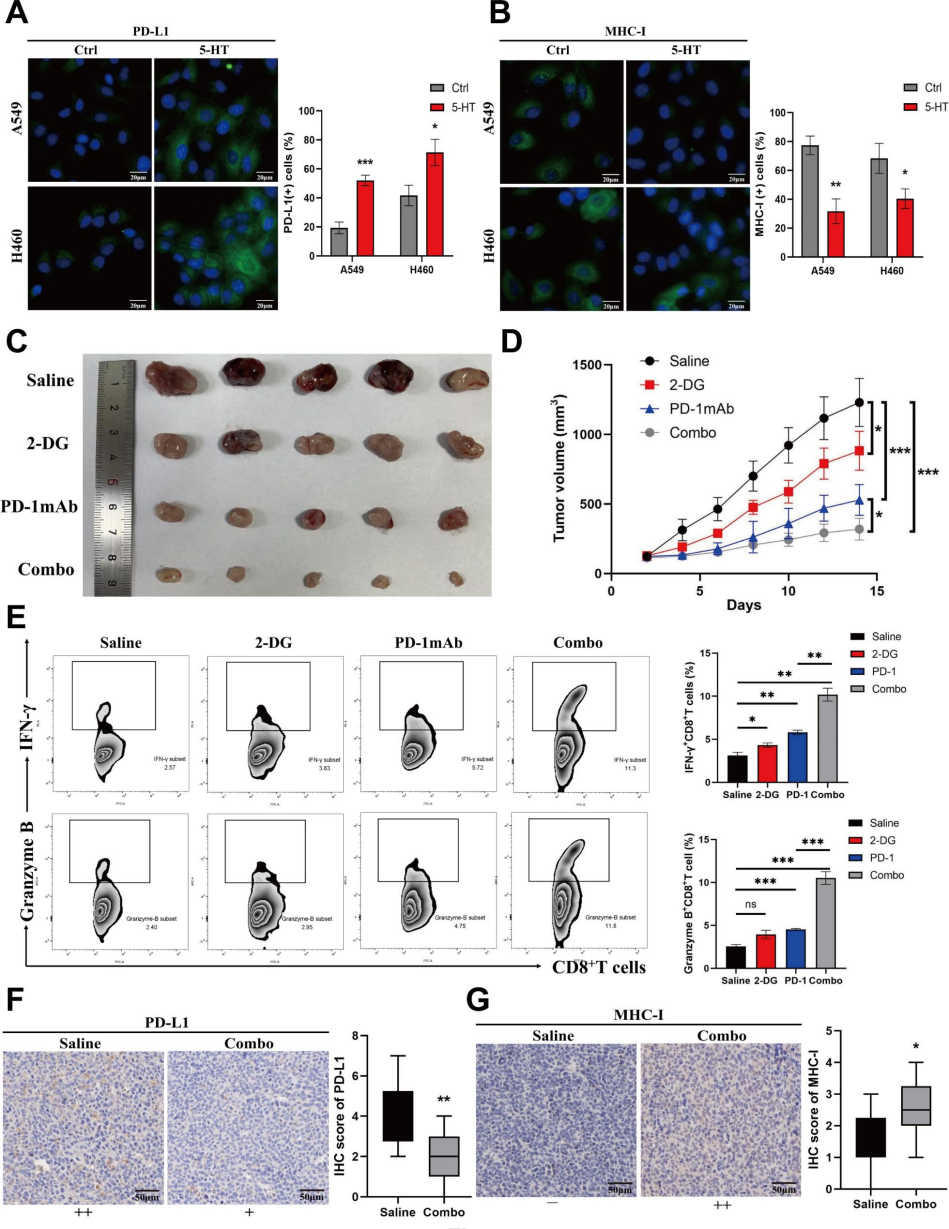
Inhibition of 5-HT-mediated tumor metabolic reprogramming can alleviate immune tolerance in mic **(A)** Immunofluorescence detection of cells indicates programmed cell death ligand 1 (PD-L1) expression. Fluorescence expression statistics are shown on the right panel. **(B)** Immunofluorescence detection of cells indicates MHC-I expression. Fluorescence expression statistics are shown on the right panel. **(C)** The mice LLC was planted into the dorsal skin of the C57 mice. When the tumor reached 100 mm^3^, 5-HT (500 µg/kg, intratumoral injection, q3d) treatment was started for all groups. After 1 week later, mice were treated in groups using 2-DG (0.03%, w/w) and PD-1mAb (10 mg/kg, ip, twice a week). When the maximum diameter of the tumor exceeded 1.5 cm, the mice were sacrificed through cervical dislocation, and the tumors were exfoliated and photographed. **(D)** The tumor volume was measured every 3 days. The difference between the mouse tumor sizes in different therapeutic groups was evaluated through the t-test with GraphPad Prism8 software. **(E)** Detection of IFN-γ and granzyme B secreted by CD8 + T cells in tumor-infiltrating lymphocytes using flow cytometry and statistical analysis (Right panel). **(F)** IHC revealed PD-L1 expression in mouse tumor tissues and the differences in expression between the Combo and Saline groups. **(G)** IHC revealed the expression of MHC-I in mouse tumor tissues and the differences in the expression between the Combo and Saline groups

We examined the effect of 5-HT on the immunogenicity of tumor cells. The results showed that 5-HT significantly upregulated the expression of the cell-surface immunosuppressive molecule PD-L1 (Fig. 7A) and downregulated that of MHC-I (Fig. 7B). To further explore whether 5-HT-mediated changes in PD-L1 and major histocompatibility complex class I (MHC-I) expression are driven by alterations in cellular metabolism, we used the glycolysis inhibitor 2-DG to inhibit the 5-HT-induced Warburg effect and monitored the resulting changes in PD-L1 and MHC-I expression. Flow cytometry analysis revealed that NSCLC cells exhibited PD-L1 upregulation and MHC-I downregulation in response to 5-HT stimulation. Inhibition of 5-HT-mediated glycolysis with 2-DG successfully reversed these effects (with statistically significant differences compared to the 5-HT group). Conversely, treatment with 2-DG alone did not significantly affect PD-L1 or MHC-I expression (see Supplementary Fig. 4A, B, Additional file 4). Subsequently, we established a mice model for subcutaneous lung cancer. Each group received an intratumoral injection of 5-HT, followed by treatment according to their respective groups. The results showed that the tumor volume of mice treated with the combination of PD-1 monoclonal antibody (mAb) and 2-DG (combo group) was significantly reduced compared with that of the single-drug (PD-1 mAb and 2-DG groups) and control (saline group) groups, showing the best therapeutic efficacy (Fig. 7C, D). Flow cytometry of CD8^+^ T cells in mouse tumor tissues revealed significantly enhanced function in the combo group compared with that in the PD-1 mAb and saline groups (Fig. 7E). Mouse tumor IHC further confirmed that combination therapy significantly downregulated PD-L1 and upregulated MHC-I expression compared with those of the control group (Fig. 7F, G). These findings substantiate that 5-HT promotes immune evasion in tumor cells by upregulating PD-L1 and downregulating MHC-I expression. The use of the glycolysis inhibitor 2-DG reverses these effects and considerably enhances the anti-tumor efficacy when combined with PD-1 monoclonal antibody therapy.

**Fig. 8:**
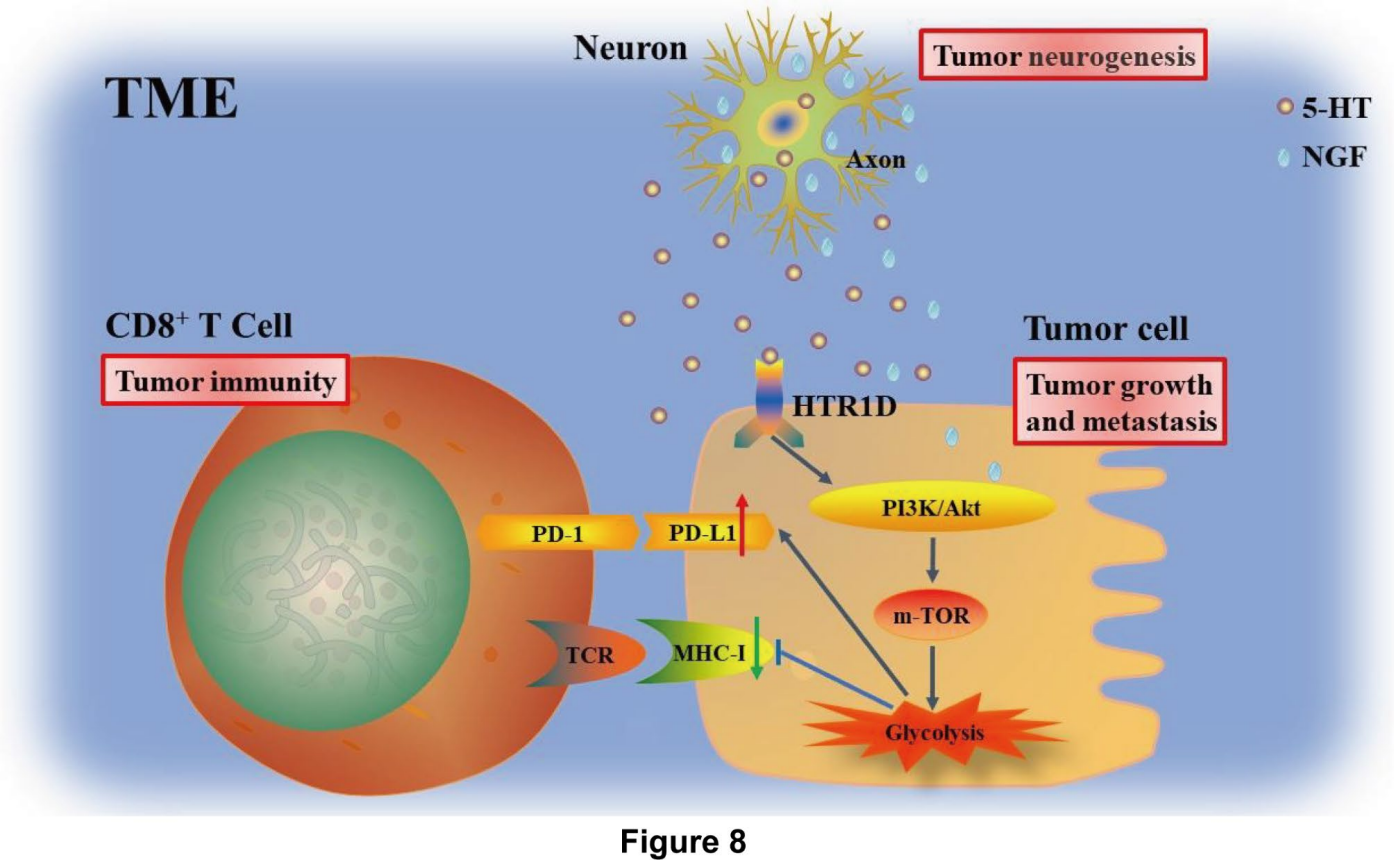
Mechanisms of the effect of neuroinvasion on immune tolerance in NSCLC NGF released from NSCLC cells induces neuronal cell differentiation and axonogenesis in the tumor microenvironment (TME). Owing to the innervation in the TME, nerve fibers release neurotransmitter 5-HT, which binds to the HTR1D receptor on the surface of NSCLC cells, activates the PI3K/Akt/mTOR pathway, enhances the Warburg effect in NSCLC cells, and induces tumor immunosuppression. Relieving tumor metabolic reprogramming-induced immunosuppression can synergistically enhance the efficacy of PD-1 monotherapy

Our findings provide compelling evidence that NGF release from NSCLC cells induces neuronal differentiation and axonogenesis in the TME. Tumor innervation causes 5-HT release from nerve fibers, which binds to NSCLC cells. This binding activates HTR1D receptors, subsequently stimulating the PI3K/Akt/mTOR pathway and enhancing the Warburg effect. This metabolic reprogramming promotes the malignant transformation of NSCLC cells and induces immunosuppression within the TME (Fig. 8).

## Discussion

Previous studies on PNI primarily described the process of tumors growing and migrating along pre-existing nerve fibers. However, recent research has uncovered a new phenomenon, where tumors are infiltrated by nerves in a manner analogous to angiogenesis, actively regulating tumor initiation and progression [28]. The significance of the TME in dynamically regulating tumor progression and influencing treatment efficacy is now recognized. Growing evidence indicates that autonomic nerves are present within the TME. Because these neural cells are located in the TME, their responses to stimuli are not solely governed by central nervous system regulation but are also strongly influenced by the surrounding environment [29]. Similarly, the response of nerve cells to stimuli can promote tumor growth and progression through the release of neurotransmitters, affecting the transcription, translation, and cytoskeletal dynamics in tumor cells [30]. In our study, IHC staining of tumor tissues from patients with NSCLC using the pan-neuronal marker PGP9.5 revealed extensive neural infiltration in NSCLC, indicating that nerve infiltration is also a pathological feature of NSCLC. Further correlation analysis confirmed that the degree of nerve infiltration was correlated with patient lymph node metastasis (*p* = 0.0017), pathological grade (*p* = 0.0262), and PD-L1 expression (*p* = 0.0201). Moreover, our retrospective clinical study supported a positive correlation between the degree of nerve infiltration and OS in patients with NSCLC (*p* = 0.0139). This suggests that the presence of nerve fibers in NSCLC predicts greater tumor metastatic potential and poorer prognosis than that of NSCLC in the absence of nerve fibers.

PC12 cells are used in neurite growth studies [31]. In this study, in vitro co-culture experiments with neuron-like PC12 cells revealed that NSCLC cells can induce axonogenesis in PC12 cells by releasing NGF. NGF is the most characterized neurotrophic factor responsible for the development of the early embryonic nervous system and is primarily responsible for the control of synaptic function and plasticity and for maintaining neuronal cell survival, morphology, and differentiation [32]. Neurotrophic factor drives axonogenesis by stimulating tyrosine kinase receptors (tyrosine kinases; Trk-A, -B, and-C) and the p75 neurotrophic factor receptor (p75 NT receptor, p75^NTR^), a member of the tumor necrosis factor receptor superfamily, in neuronal cells [33]. p75^NTR^ has the same affinity for all NTs, whereas TrkA binds preferentially to NGF; TrkB binds preferentially to BDNF and neurotrophin-4 (NT-4), and TrkC binds preferentially to neurotrophin-3 (NT-3) [34, 35]. While our results indicate that the expression of other neurotrophic factors is also elevated in NSCLC cells, the observed upregulation of the TrkA receptor expression in PC12 cells within the co-culture system suggests its direct correlation with the increased the increased NGF levels in the supernatant. Following the introduction of the NGF inhibitor Ro 08-2750 into the co-culture system, we observed a significant suppression of axonal growth in neuronal cells. These findings further confirm that NGF, rather than other neurotrophic factors, plays a primary role in driving neural infiltration in NSCLC. Consistently, in prostate, gastric, and pancreatic cancers, increased nerve density is reportedly correlated with increased tumor aggressiveness in the periphery and within tumors [14, 15, 36]. Singh et al. demonstrated that TrkA, the NGF receptor, acts as a tumor necrosis factor receptor-associated factor 4 (TRAF4)-targeted ubiquitination substrate to stimulate p38 MAPK activation and invasion-related gene expression and that inhibition of TrkA activity eliminated TRAF4-dependent prostate cancer cell invasion [37]. These results suggest a potential tumor-promoting effect on nerves. Similarly, we observed the pro-proliferative, invasive, and migratory effects of neural cells on NSCLC cells at the cellular level and validated them at the animal level. An in-situ nerve infiltration lung cancer model was constructed by administering NGF to mice, and IHC was performed on the tumor tissues of the mice, which revealed that tumor Ki-67 and N-cadherin expression were significantly upregulated in the nerve infiltration model group compared with that in the normal group. N-cadherin is a key adherens junction protein that plays a pivotal role in cell-cell adhesion and has been implicated in various aspects of tumor biology, including metastasis [38]. The use of N-cadherin to elucidate tumor metastasis capabilities is based on its well-established role in facilitating epithelial-to-mesenchymal transition (EMT), a critical process in tumor progression and metastasis [39]. These findings suggest that nerve infiltration is significant in controlling the occurrence and progression of NSCLC.

Neurotransmitters are the link between nerves and tumor cells in the TME. Neurotransmitters released from nerve fibers in the TME can play various regulatory roles by binding to specific neurotransmitter receptors on tumor cells and activating inhibitory or stimulatory signaling pathways [40]. The levels of 5-HT were significantly increased in the tumor tissues of mice in the high-nerve infiltration group. The IHC of serial sections of tumor tissues revealed concordance between the two expression levels. These results suggest that neural infiltration in tumors promotes tumor progression by releasing the neurotransmitter 5-HT, prompting further investigation of the biological function of 5-HT in NSCLC.

Zhu P et al. reported that serotonergic neurons in the intestines promote colorectal carcinogenesis by producing 5-HT to initiate the self-renewal of colonic stem cells [41]. Gautam J et al. reported that autocrine 5-HT promotes invasiveness and angiogenesis in triple-negative breast cancer [42]. In gastric cancer, 5-HT activation of the PI3K/Akt/mTOR signaling pathway induces increased expression of HIF1α and ABCD1, enhanced viability of gastric adenocarcinoma cells under metabolic stress, reduced cellular and lipid reactive oxygen clusters, and inhibited iron-dependent cell death [43]. These findings suggest that 5-HT activates multiple signaling pathways that regulate the malignant phenotype of tumors. Our results are consistent with these findings, stating that 5-HT promotes the proliferation, invasion, and metastatic ability of NSCLC cells and is a crucial molecule that mediates NSCLC progression. The function of 5-HT is primarily mediated by activation of the 5-HT receptor (HTR), which contains seven families ranging from HTR1 to HTR7, of which there are 15 subunits depending on conformation. Except for HTR3, a ligand ion-gated channel, all receptors are G protein-coupled (GPCR) [44]. Thus, the 5-HTR diversity suggests many functional differences among the receptors. By assessing the expression of 5-HTR mRNA in NSCLC cells after treatment with 5-HT, the critical role of HTR1D in promoting NSCLC progression via 5-HT was determined. Moreover, by constructing NSCLC cell lines with a stable HTR1D knockdown, we found that the tumor-promoting effect of 5-HT was not exerted in cells with deficient HTR1D expression.

To elucidate the potential mechanisms by which 5-HT exerts its tumor-promoting effects, the whole-genome transcriptome of NSCLC cells after 5-HT stimulation was sequenced and analyzed. These results suggest that the ability of 5-HT to promote tumor progression depends on the Warburg effect and is accompanied by the activation of the hypoxic state. Indeed, over a century ago, Otto Warburg proposed that tumor cells derive energy predominantly through glycolysis rather than OXPHOS, marking the glycolytic phenotype as a widely studied hallmark of cancer [45]. Alterations in energy metabolism are a “biochemical fingerprint” of tumor cells and one of the hallmarks of cancer, characterized by the preferential use of glycolysis to provide energy within tumor cells, independent of oxygen [23]. The alteration in cellular energy metabolism depends on the regulation of the transcription levels of glycolytic rate-limiting enzymes. We observed the upregulation of several glycolysis-related enzymes at the mRNA level, and, guided by transcriptomic sequencing, prioritized the rate-limiting enzyme HK2 and hypoxia-inducible factor HIF-1α for protein-level validation. Our findings indicate that the metabolic reprogramming induced by 5-HT depends on the coordinated upregulation of both HK2 and HIF-1α. This metabolic reprogramming drives tumor cells to sustain their elevated energy, biosynthesis, and redox needs by increasing glucose uptake and lactate production [46]. PI3K/Akt cascade signaling reportedly regulates vital metabolic processes, including glucose metabolism, macromolecular biosynthesis, and maintenance of redox homeostasis to support metabolic homeostasis and cellular growth and metabolism [47]. Moreover, mTOR is an intracellular energy-sensing molecule, and its energy metabolic adaptation influences c-Myc and HIF-1α transcription, regulating the expression of enzymes related to glycolytic metabolism [45, 48]. In our study, the specific inhibitors LY294002 and rapamycin were used to target PI3K and mTOR pathways after 5-HT stimulation. These results suggest that inhibiting the PI3K/Akt/mTOR signaling pathway impairs the 5-HT-mediated glycolytic phenotype. In contrast, in HTR1D-knockout NSCLC cells, 5-HT caused no changes in the phosphorylation levels of PI3K, Akt, or mTOR, which are critical downstream molecules of the G protein. Therefore, we suggest that under metabolic stress, NSCLC cells expressing HTR1D utilize more glucose to gain a growth advantage through the 5-HT-mediated Warburg effect and control cell metabolic reprogramming by activating the PI3K/Akt/ mTOR signaling pathway. Cascone et al. concluded that highly glycolytic tumor cells can mediate the inhibition of T cell killing and trafficking in the TME, causing resistance to overt T cell therapy [49]. Similarly, Hao et al. showed that increased glycolysis in tumor cells mediates T-cell hyporesponsiveness in cancer [50]. These observations indicate that tumor glycolysis leads to the development of a highly suppressive immune microenvironment. Consequently, metabolic alterations in tumor cells may result in changes in the TME that impact tumor immunity, promoting immune tolerance and immune evasion by tumor cells.

Multiple drug resistance in lung cancer causes poor treatment efficacy and is considered the current focus of tumor immunotherapy [51]. PD-L1 and MHC-I are significant immunoregulatory molecules on the surface of tumor cells. PD-L1 is expressed on the surface of tumor cells and induces inhibitory signals to suppress tumor-killing activity by binding to the PD-1 receptor on the surface of CD8^+^ T cells. MHC-I acts as an antigen-presenting molecule that presents tumor antigens to CD8^+^ T cells, exerting antitumor immune responses [52]. ICI therapy targeting the tumor immune checkpoint, PD-L1, has recently overcome the limitations of traditional chemotherapy in treating tumors. However, many tumors evade immune recognition and killing by inhibiting or downregulating MHC-I expression, resulting in a weak therapeutic response to ICIs. For example, in patients with pancreatic cancer, the glucocorticoid receptor (GR) was associated with high PD-L1 expression, low MHC-I expression, and poor survival, whereas depletion of tumor-specific GR or pharmacological inhibition of GR-sensitized pancreatic cancer in mice was associated with immunotherapy [53]. Our results suggest that 5-HT exacerbates tumor immunosuppression in the TME by affecting tumor cell metabolic reprogramming, downregulating tumor cell MHC-I, and upregulating PD-L1 while reducing tumor immunogenicity. In vivo, blocking 5-HT-mediated metabolic reprogramming-induced immunosuppression in tumors significantly enhanced the function of CD8^+^ T-cells in mouse tumors. This synergistically enhances the efficacy of PD-1 mAb immunotherapy to achieve better tumor control.

Although this study highlights the pivotal role of autonomic nerves and neurotransmitters in the TME during NSCLC progression, several limitations should be noted. HK2 was validated at the protein level due to its central role in glycolysis and consistency with our transcriptomic data, whereas other glycolytic enzymes were assessed only at the mRNA level. Extending protein-level validation for these enzymes could provide additional insights. The intricate neuro-humoral regulatory network also implies that other neurotrophic factors and neurotransmitters, potentially playing crucial roles, should not be overlooked. Additionally, the retrospective clinical study in this research only established correlations between neural pathways and tumor progression without demonstrating causation. Therefore, further prospective studies are required to confirm the clinical significance and broader applicability of these findings.

## Conclusions

The mutual crosstalk between nerves and NSCLC cells described in this study significantly enhances our understanding of the nerve-tumor-immunoregulatory network within the TME. This study is the first time to establish a direct connection between nerve infiltration, NSCLC progression, and immunosuppression, thereby providing a solid theoretical foundation for improving tumor immunotherapy efficacy. These insights deepen our comprehension of the neural-tumor-immunoregulatory regulatory network in the TME, facilitating the diagnosing and monitoring of tumor progression and highlighting the potential of targeting neural infiltration as a therapeutic approach. Understanding the mechanisms of neural infiltration and its effects on tumor progression and immune response could lead to the identification of novel biomarkers for early detection and prognosis. Moreover, this study paves the way for exploring combination therapies that integrate neural targeting with existing immunotherapies, potentially enhancing patient outcomes and opening new avenues for personalized cancer treatment.

## Electronic supplementary material

Below is the link to the electronic supplementary material.

Supplementary Material 1

Supplementary Material 2

Supplementary Material 3

Supplementary Material 4

Supplementary Material 5

## Data Availability

All datasets and materials generated for this study are included in the manuscript or supplementary material in additional files.

## Abbreviations

TME: Tumor microenvironment
NSCLC: Non-small cell lung cancer
NGF: Nerve growth factor
PC12: Pheochromocytoma cells
PD-LI: Programmed cell death ligand
PDAC: Pancreatic ductal adenocarcinoma
5-HT: 5-hydroxytryptamine
2-DG: 2-deoxy-D-glucose
LLC: Lewis lung cancer cell
OCR: Oxygen consumption rate
ECAR: Extracellular acidification rate
PGP9.5: Protein gene product 9.5
OS: Overall survival
BDNF: Brain-derived neurotrophic factor
DEGs: Differentially expressed genes
OXPHOS: Oxidative phosphorylation
mAb: Monoclonal antibody
Trk: Tyrosine kinase
TRAF4: Tumor necrosis factor receptor-associated factor 4
HTR: 5-HT receptor
GR: Glucocorticoid receptor
