# Supplementary material for: Mechanisms of neural infiltration-mediated tumor metabolic reprogramming impacting immunotherapy efficacy in non-small cell lung cancer
Source: J Exp Clin Cancer Res. 2024 Oct 10;43:284. doi: 10.1186/s13046-024-03202-9 (PMC11465581; doi:10.1186/s13046-024-03202-9)
Supplement: Supplementary file 1 — Supplementary Material 1 [file 13046_2024_3202_MOESM1_ESM.docx]

**
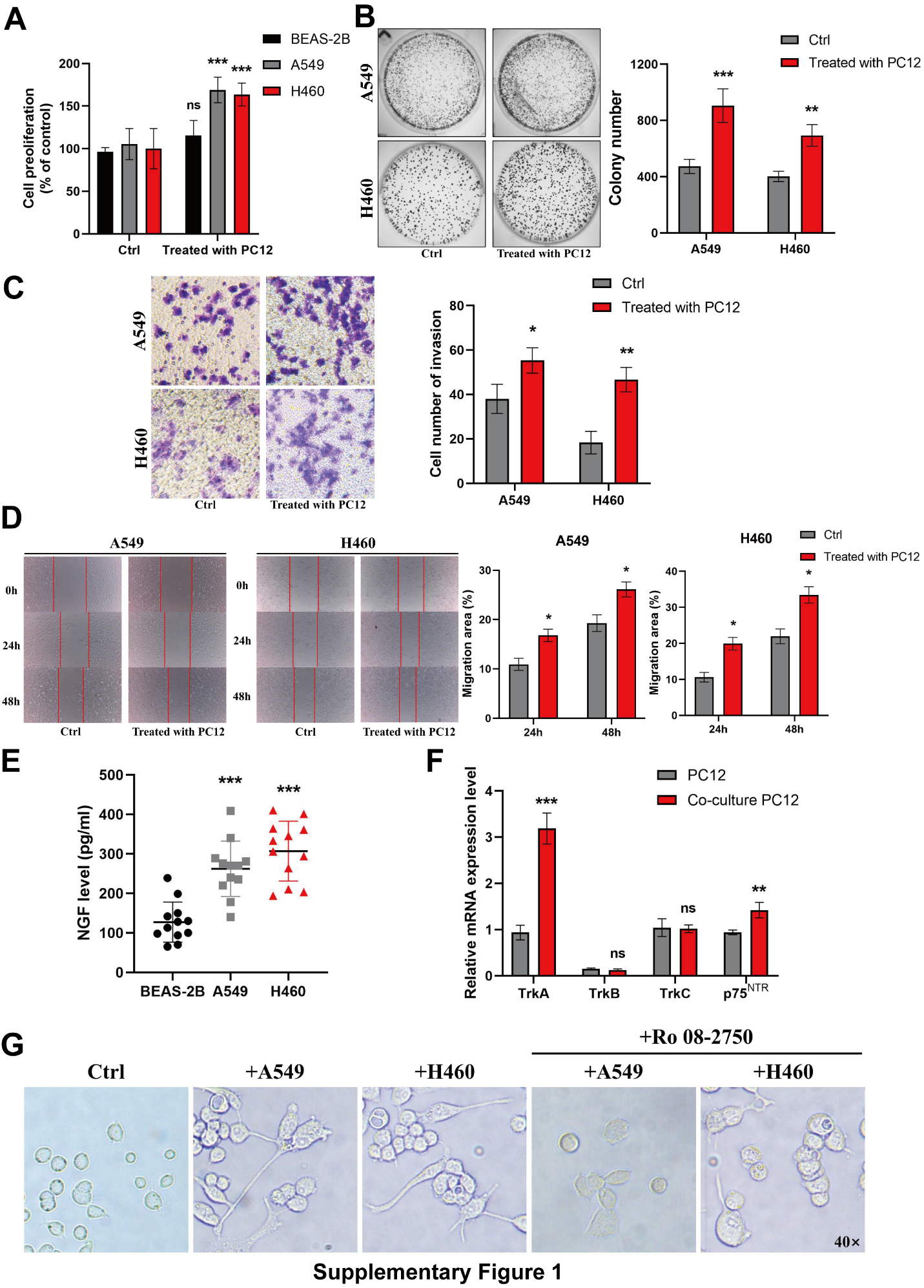
Supplementary Fig. 1**

(A) Effect of co-culture conditions with PC12 cells on the proliferative capacity of A549, H460, and BEAS-2B cells. (B) Effect of co-culture conditions with PC12 cells on colony formation by NSCLC cell lines. Statistical analyses were performed on the right side. (C) Effect of co-culture conditions with PC12 cells on the invasive ability of NSCLC cell lines, with statistical analyses on the right-hand side. (D) Effect of co-culture conditions with neural cells on the migratory ability of NSCLC cell lines, with statistical analyses on the right-hand side. (E) NGF levels in the supernatants of A549, H460, and BEAS-2B cells were measured using ELISA. (F) Neuroreceptor expression changes at the mRNA level in PC12 cells under co-culture conditions with NSCLC cell lines. (G) Neurotrophic effects of the NGF inhibitor (Ro 08-2750) on neurotrophic effects in NSCLC cell lines.
