# Supplementary material for: Mechanisms of neural infiltration-mediated tumor metabolic reprogramming impacting immunotherapy efficacy in non-small cell lung cancer
Source: J Exp Clin Cancer Res. 2024 Oct 10;43:284. doi: 10.1186/s13046-024-03202-9 (PMC11465581; doi:10.1186/s13046-024-03202-9)
Supplement: Supplementary file 2 — Supplementary Material 2 [file 13046_2024_3202_MOESM2_ESM.docx]

**
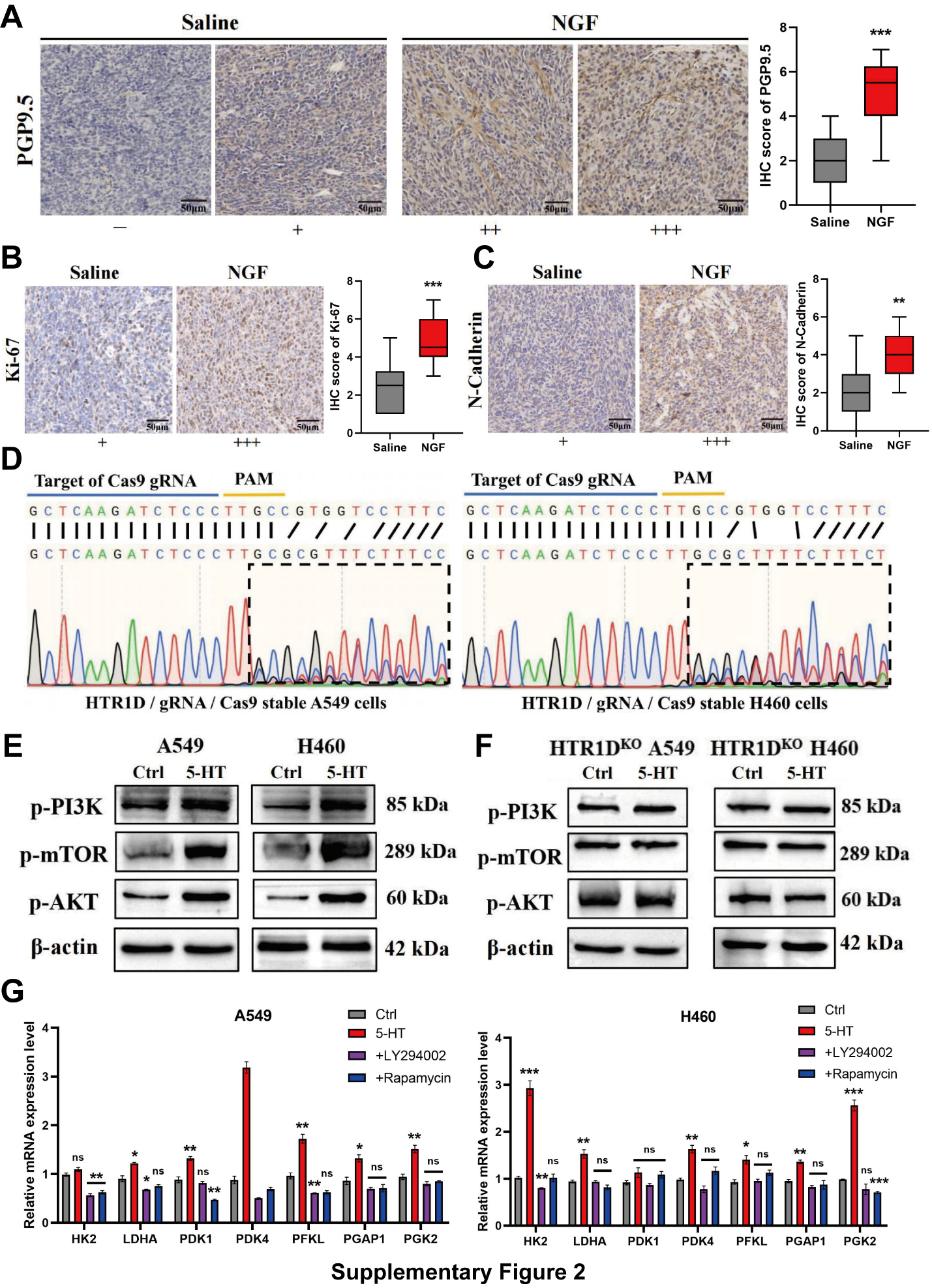
Supplementary Figure 2.**

(A) Representative results and statistical plots of PGP9.5 staining in the tumors of the two mouse groups. (B) Representative results of Ki-67 staining and statistical plots of tumor expression in the two mouse groups. (C) Representative results of N-cadherin staining and statistical plots of tumor expression in the two mouse groups. (D) DNA from stabilized HTR1D-knockout cells was extracted for sequencing to validate the HTR1D^KO^ NSCLC cell lines. (E) The effect of 5-HT stimulation on protein phosphorylation levels in the PI3K/Akt/mTOR signaling pathway in normal and HTR1D^KO^ NSCLC cell lines. (F) Changes in the mRNA expression levels of 5-HT-mediated glycolysis-related enzymes induced by LY294002 and Rapamycin.
