# Supplementary material for: Mechanisms of neural infiltration-mediated tumor metabolic reprogramming impacting immunotherapy efficacy in non-small cell lung cancer
Source: J Exp Clin Cancer Res. 2024 Oct 10;43:284. doi: 10.1186/s13046-024-03202-9 (PMC11465581; doi:10.1186/s13046-024-03202-9)
Supplement: Supplementary file 3 — Supplementary Material 3 [file 13046_2024_3202_MOESM3_ESM.docx]

**
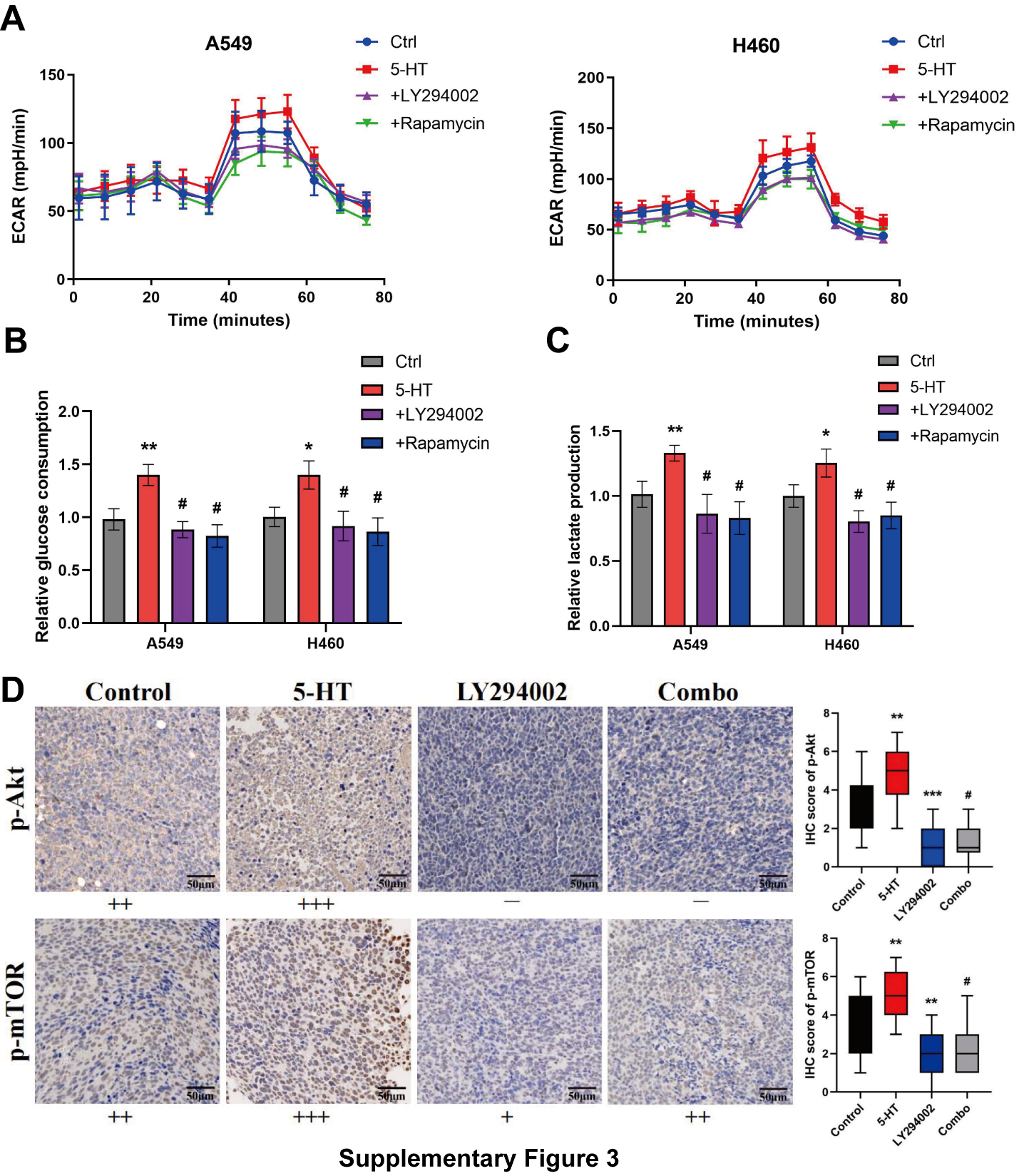
Supplementary Figure 3.**

(A) Changes in the ratio of 5-HT-mediated extracellular acidification induced by LY294002 and Rapamycin treatment. (B) Changes in 5-HT-mediated glucose depletion induced by LY294002 and Rapamycin treatment. (C) Changes in 5-HT-mediated lactate levels induced by LY294002 and Rapamycin treatment. (D) Representative results and expression statistics of p-AKT and p-mTOR in mouse tumor tissues. * Indicates statistically significant difference of p < 0.05 when compared with the control group; # Indicates statistically significant difference of p < 0.05 when compared with the 5-HT group.
