# Supplementary material for: Mechanisms of neural infiltration-mediated tumor metabolic reprogramming impacting immunotherapy efficacy in non-small cell lung cancer
Source: J Exp Clin Cancer Res. 2024 Oct 10;43:284. doi: 10.1186/s13046-024-03202-9 (PMC11465581; doi:10.1186/s13046-024-03202-9)
Supplement: Supplementary file 4 — Supplementary Material 4 [file 13046_2024_3202_MOESM4_ESM.docx]

**
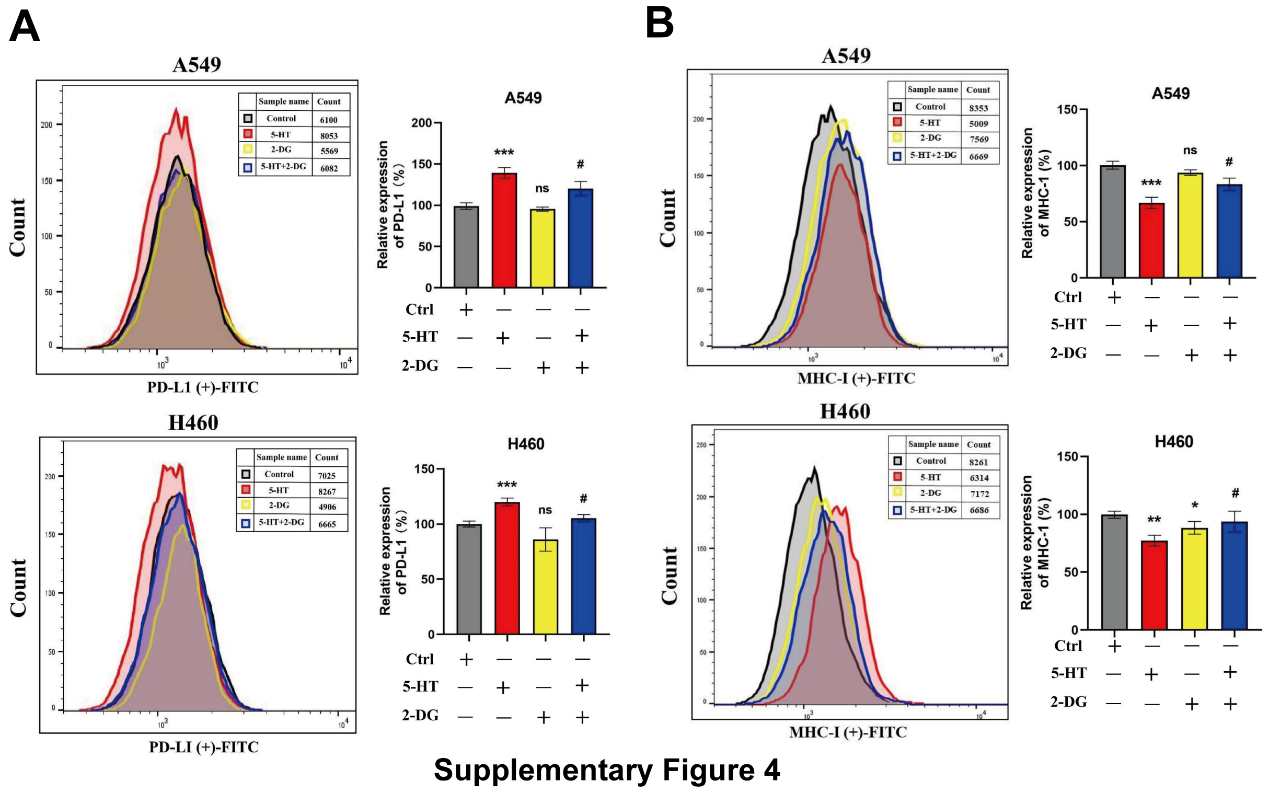
**

**Supplementary Figure 4.**

(A) Flow cytometry detection of the effect of 2-DG on 5-HT-mediated changes in cell surface PD-L1 expression. (B) Flow cytometry detection of 2-DG on 5-HT-mediated changes in cell surface MHC-I expression. * Indicates statistically significant difference of p < 0.05 compared with the control group; # Indicates statistically significant difference of p < 0.05 compared with the 5-HT group.
