# Supplementary figures and images for: Mechanisms of neural infiltration-mediated tumor metabolic reprogramming impacting immunotherapy efficacy in non-small cell lung cancer
Source: J Exp Clin Cancer Res. 2024 Oct 10;43:284. doi: 10.1186/s13046-024-03202-9 (PMC11465581; doi:10.1186/s13046-024-03202-9)

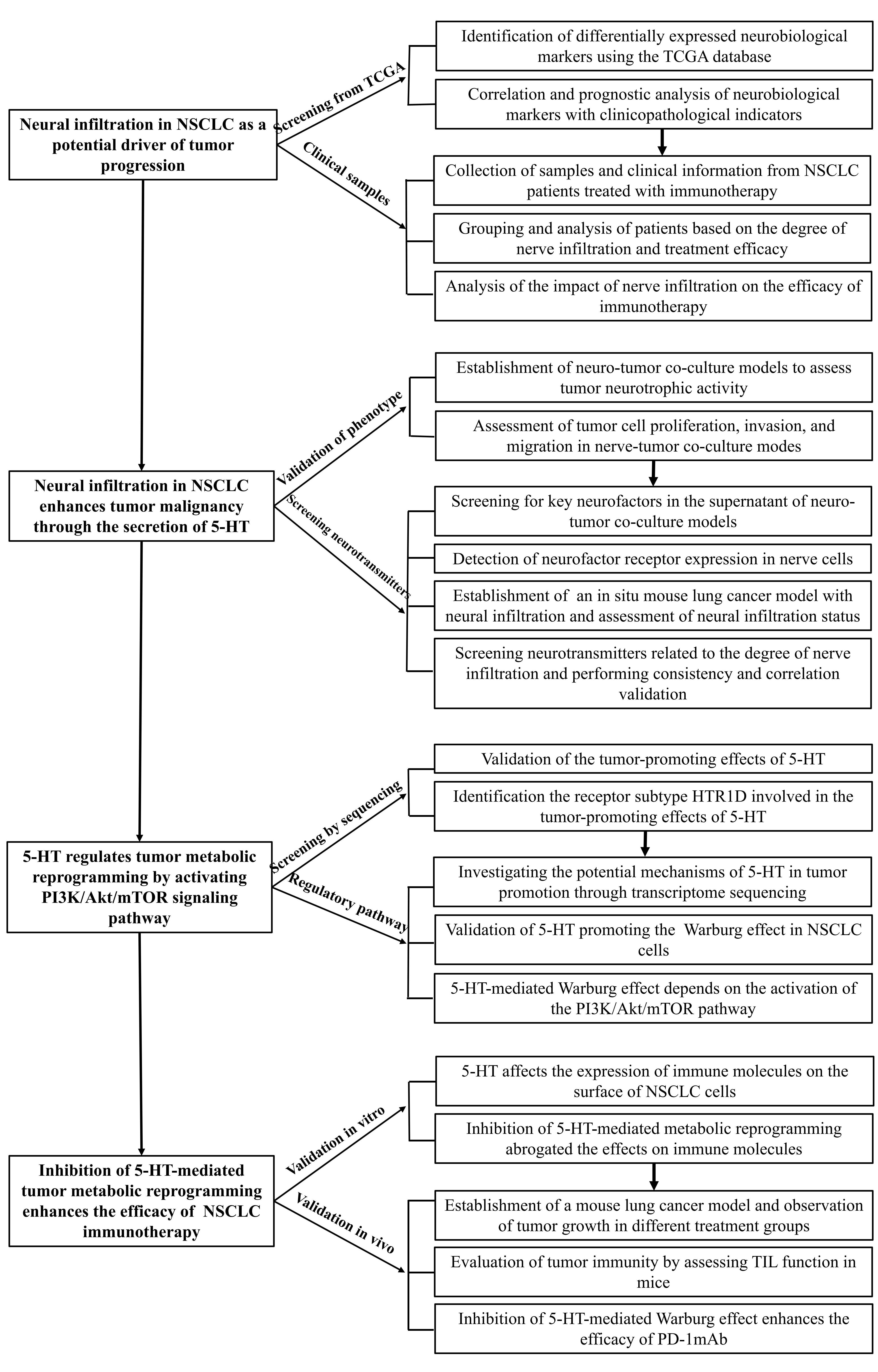

Supplement: Supplementary file 5 — Supplementary Material 5 [file 13046_2024_3202_MOESM5_ESM.jpg]
